# Supplementary material for: Evaluation of synthetic mRNA with selected UTR sequences and alternative poly(A) tail, in vitro and in vivo
Source: Mol Ther Nucleic Acids. 2025 Jul 30;36(3):102648. doi: 10.1016/j.omtn.2025.102648 (PMC12355064; doi:10.1016/j.omtn.2025.102648)
Supplement: Document S1. Figures S1–S11 and Tables S1–S4 [file mmc1.pdf]

OMTN, Volume *36*

Supplemental information

Evaluation of synthetic mRNA with selected UTR sequences and alternative poly(A) tail, *in vitro* and *in vivo*

Ayoub Medjmedj, Hugo Genon, Dounia Hezili, Albert Ngalle Loth, Rudy Clemençon, Cyril Guimpied, Lucile Mollet, Anne Bigot, Frank Wien, Josef Hamacek, Clément Chapat, and Federico Perche

Table S1: *sequences of the poly(A) tails evaluated.*

Name	Sequence
A120	AAAAAAAAAAAAAAAAAAAAAAAAAAAAAAAAAAAAAAAAAAAAAAAAAAAAAAAAAAAAA<br>AAAAAAAAAAAAAAAAAAAAAAAAAAAAAAAAAAAAAAAAAAAAAAAAAAAAAAAAAAA
A30L70	AAAAAAAAAAAAAAAAAAAAAAAAAAAAAAGCATATGACTAAAAAAAAAAAAAAAAAAAAAA<br>AAAAAAAAAAAAAAAAAAAAAAAAAAAAAAAAAAAAAAAAAAAAAAAAAA
A/G	AAAAAAAAAAGAAAAAAAAAAGAAAAAAAAAAGAAAAAAAAAAGAAAAAAAAAAGAAAAAA<br>AAAAGAAAAAAAAAAGAAAAAAAAAAGAAAAAAAAAAGAAAAAAAAAAGAAAAAAAAAAGA<br>AAAAAAAAAG

Table S2: *dsRNA levels of different IVT mRNA: All mRNAs are capped with ARCA, except for the Oz Bioscience mRNA, which is cap1.*

5'UTR	CDS	3'UTR	dsRNA % ± SD
hAg	Nano luciferase	AES	0.361 ± 0.144
		VP6	0.510 ± 0.219
		SOD	0.280 ± 0.013
UTR3		AES	0.294 ± 0.072
		VP6	0.233 ± 0.020
		SOD	0.209 ± 0.011
UTR4		AES	0.331 ± 0.147
		VP6	0.291 ± 0.079
		SOD	0.206 ± 0.008
hAg	Firefly luciferase	AES	0.431 ± 0.117
		VP6	0.379 ± 0.184
		SOD	0.259 ± 0.025
UTR3		AES	0.239 ± 0.003
		VP6	0.293 ± 0.014
		SOD	0.302 ± 0.105
UTR4		AES	0.263 ± 0.028
		VP6	0.407 ± 0.095
		SOD	0.276 ± 0.006
Firefly luciferase Oz Bioscience			0.196 ± 0.036
hAg	Spike	AES	0.175 ± 0.018
		VP6	0.163 ± 0.024
		SOD	0.142 ± 0.004

***Table S3**: dsRNA level of IVT mRNA using different cap, U or m1Ψ, and before/after oligo dT purification.*

Cap	Nucleotides	dsRNA % ± SD<br>Without Oligo dT purification	dsRNA % ± SD<br>With Oligo dT purification
None	U	0.365 ± 0.063	0.154 ± 0.008
	m1Ψ	0.288 ± 0.115	0.129 ± 0.013
ARCA	U	0.947 ± 0.112	0.552 ± 0.144
	m1Ψ	0.112 ± 0.004	0.118 ± 0.013
CleanCap AG	U	0.544 ± 0.089	0.247 ± 0.015
	m1Ψ	0.105 ± 0.004	0.140 ± 0.014

***Table S4**: qPCR primers*

	Sequences (5' -> 3')<br>FW	Sequences (5' -> 3')<br>RV
IFN-α	AAGCCATCCTTGTGCTAAGAGA	AGCAAGTTGGTTGAGGAAGAGA
IFN-β	AGCTCCAAGAAAGGACGAACA	GCCCTGTAGGTGAGGTTGAT
TNF-α	ATGAGCACAGAAAGCATGA	AGTAGACAGAAGAGCGTGGT
IL-6	CCTCTGGTCTTCTGGAGTACC	ACTCCTTCTGTGACTCCAGC
Nluc	GGAGGTGTGTCCAGTTTGTTTCAG	CACAGGGTACACCACCTTAA
GAPDH	GCTGCGTTTTACACCCTTTC	GTTTGCTCCACCCAACTGC

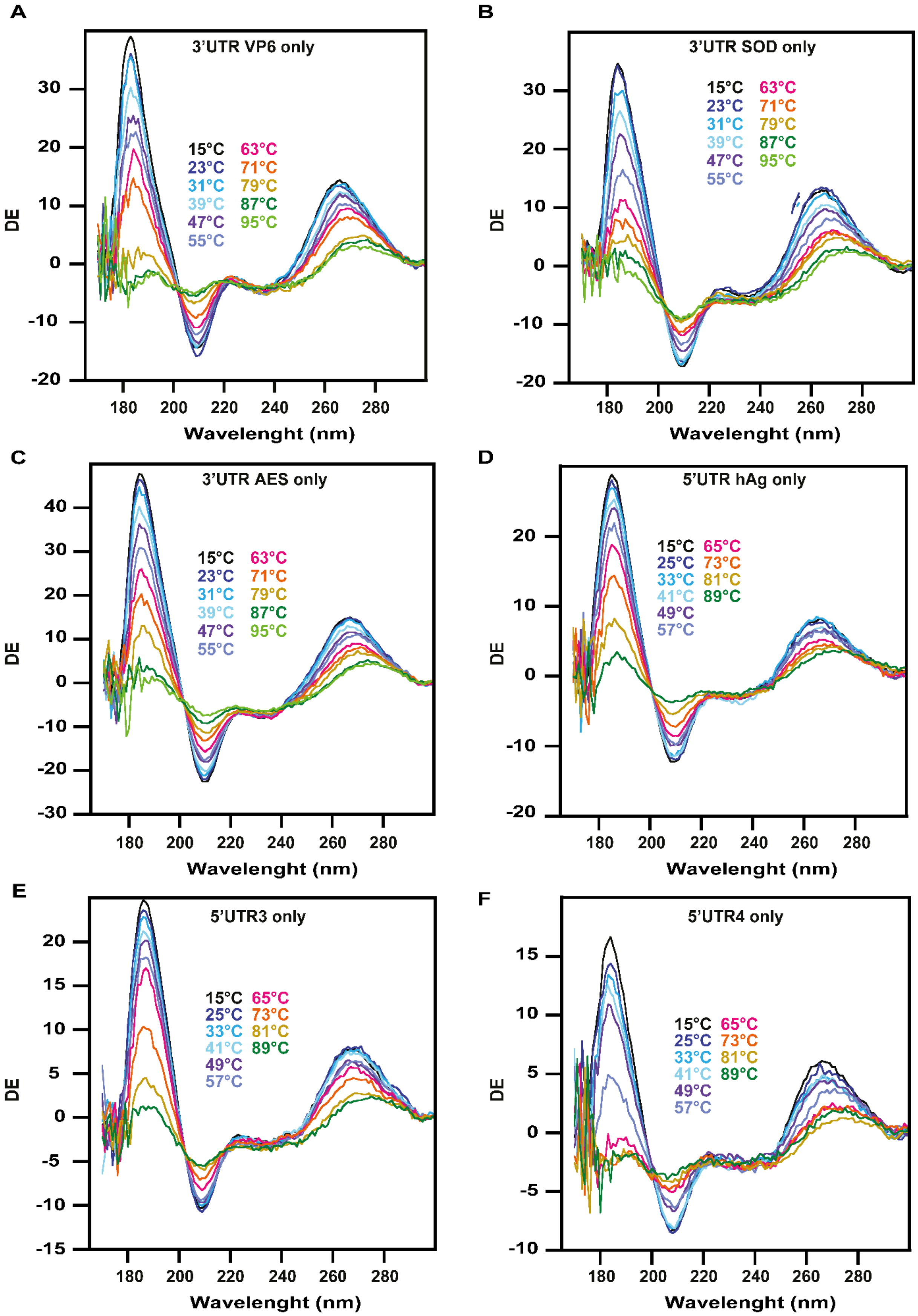

Figure S1: *Synchrotron Radiation Circular Dichroism analysis of UTR sequences. (A) 3'UTR VP6, (B) 3'UTR SOD, (C) 3'UTR AES, (D) 5'UTR hAg, (E) 5'UTR3, (F) 5'UTR4. DE: Delta Epsilon*

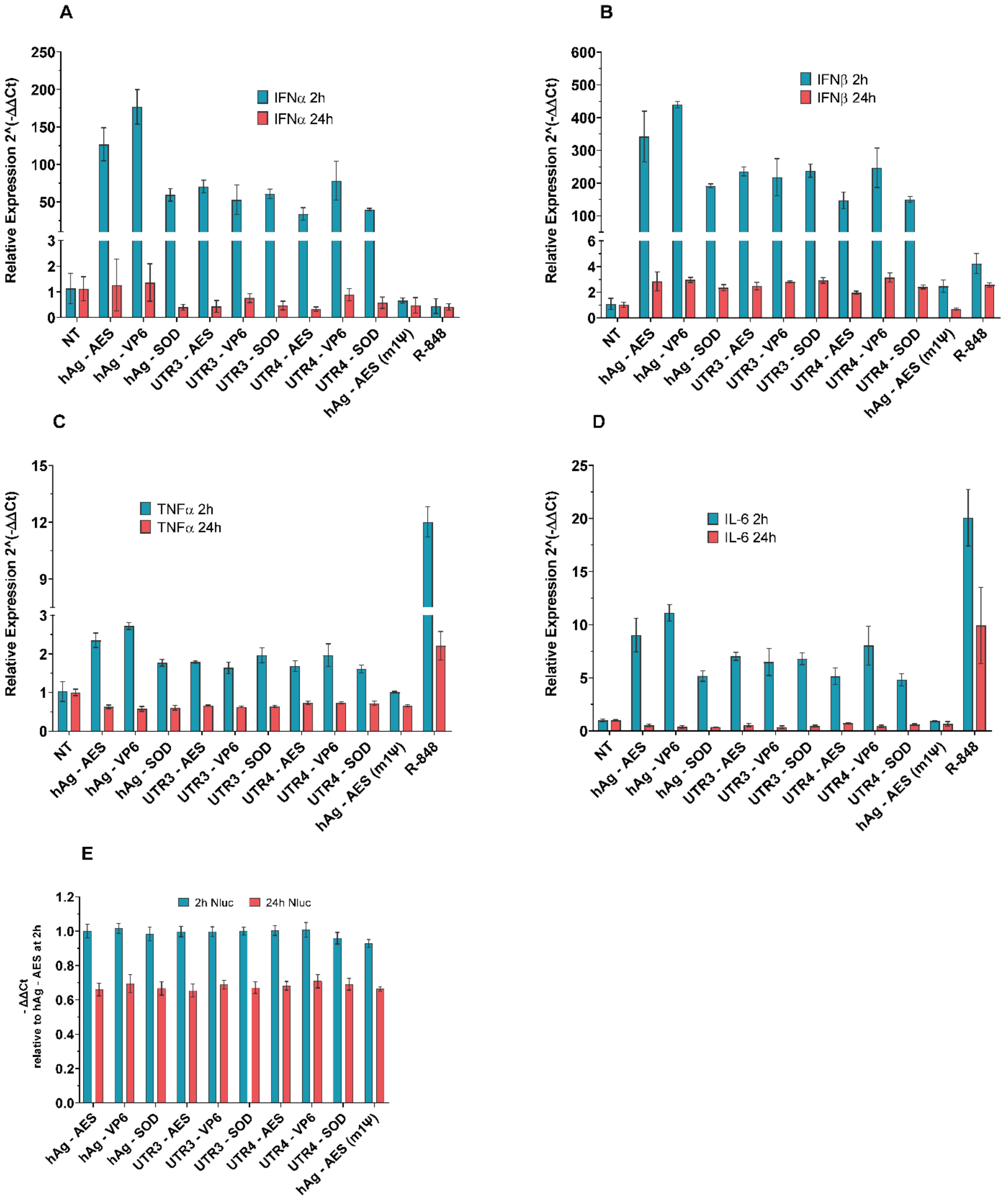

Figure S2: *qPCR results of the immunogenicity of the IVT mRNA in DC 2.4 after transfection. Results show moderate and transient activation of the interferon pathway at 2h post transfection before returning to basal levels at 24h post transfection.*

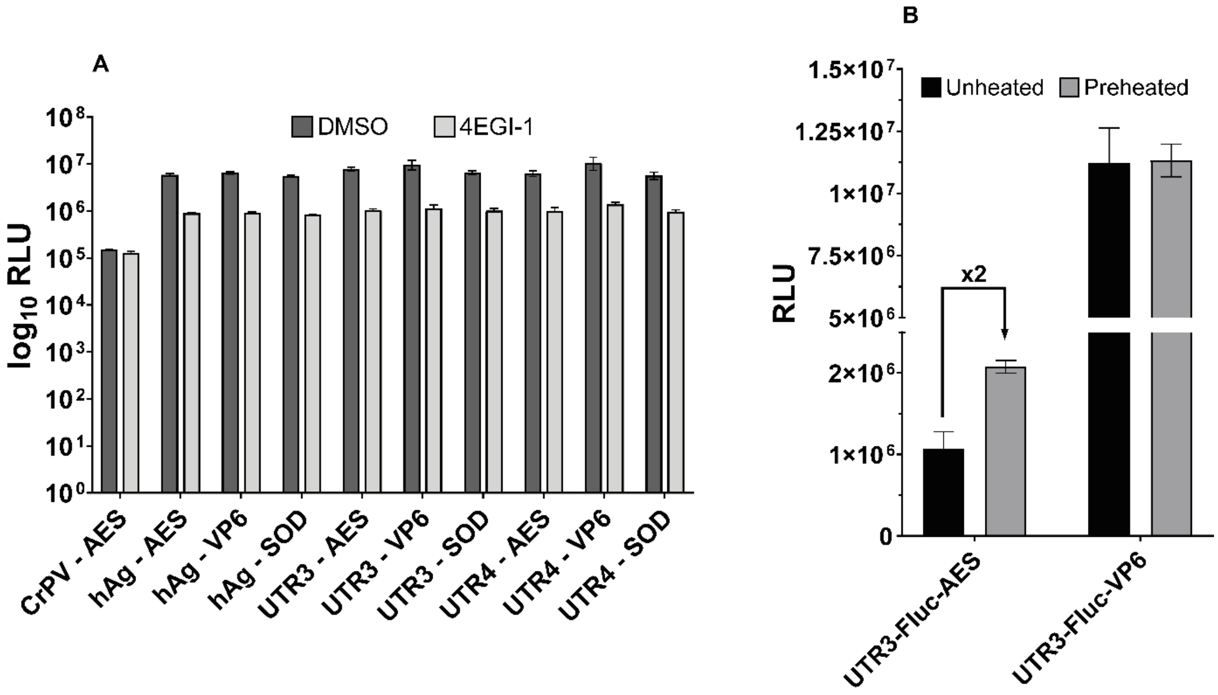

Figure S3: *In vitro translation in the rabbit reticulocytes lysate (RRL): A) Nano luciferase B) Firefly luciferase*

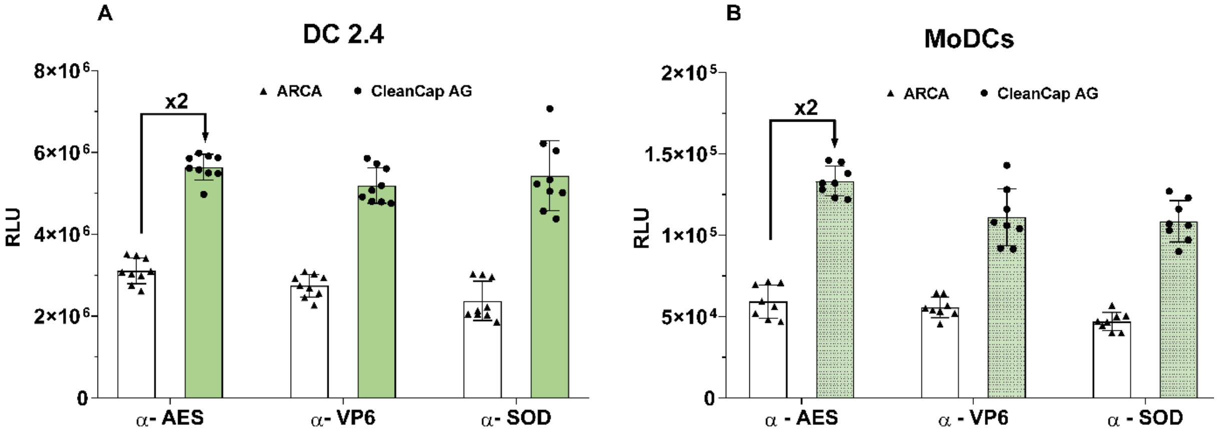

Figure S4: *ARCA-capped mRNA compared to CleanCap AG-capped mRNA in DC 2.4 cells and MoDCs primary cells*

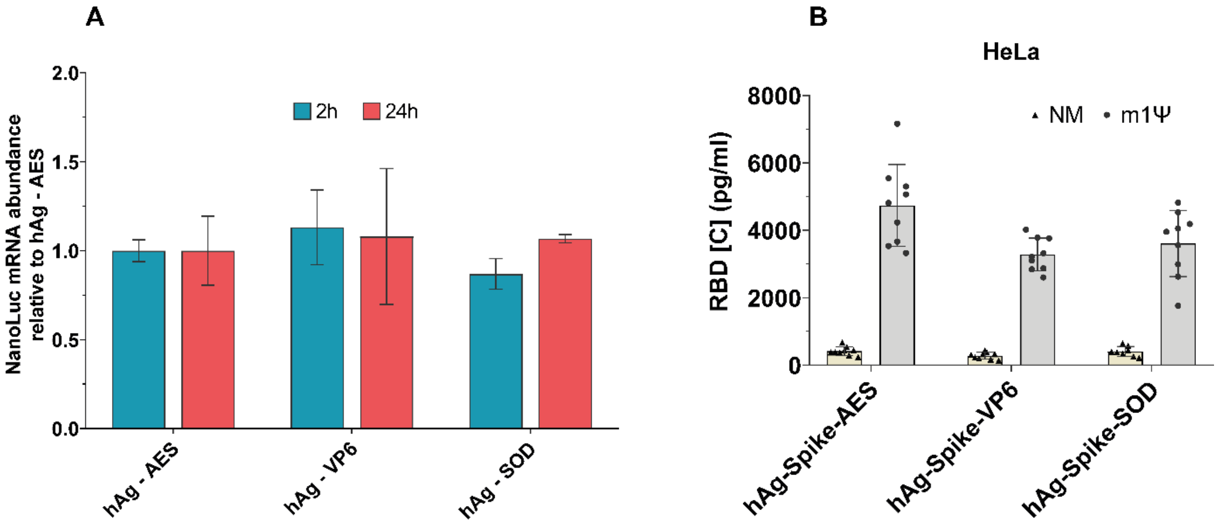

Figure S5: *A) FISH-Flow results of Nano luciferase mRNA, B) non modified spike mRNA compared to m1Ψ spike mRNA in HeLa cells 24 hours after transfection.*

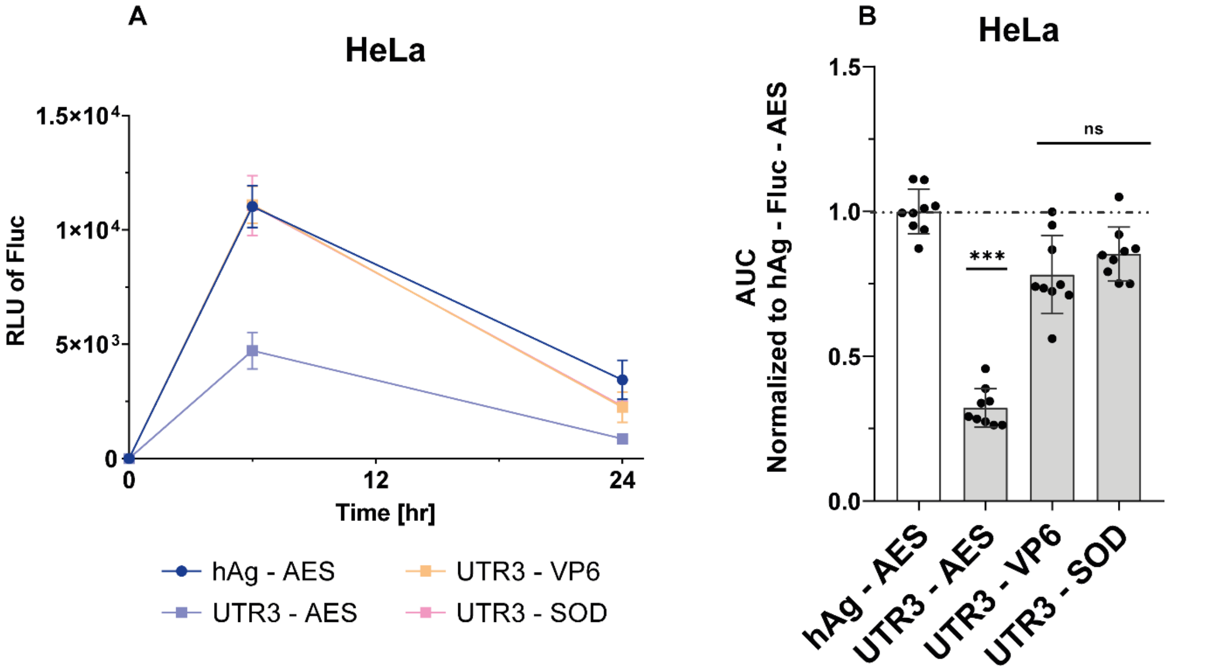

Figure S6: *Evaluation of 5'UTR3 combinations in HeLa cells: (A) Time course analysis of Fluc expression in HeLa cells transfected with the three mRNA constructs and the benchmark mRNA. Bioluminescence was acquired at 6, and 24h post-transfection, and results are expressed as mean ± SD. (B) Total protein expression over time (area under the curve) calculated from time course data and normalized to the hAg 5'UTR-AES 3'UTR.*

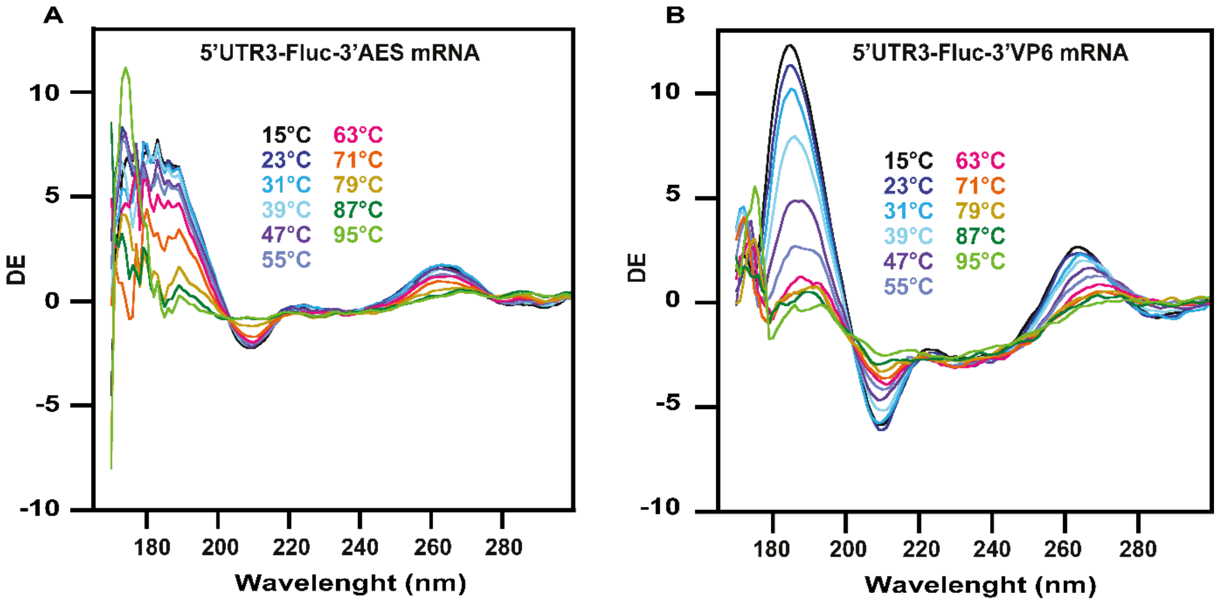

Figure S7: *Synchrotron Radiation Circular Dichroism analysis of (A) 5'UTR3-Fluc-3'AES mRNA and (B) 5'UTR3-Fluc-3'VP6 mRNA. DE: Delta Epsilon*

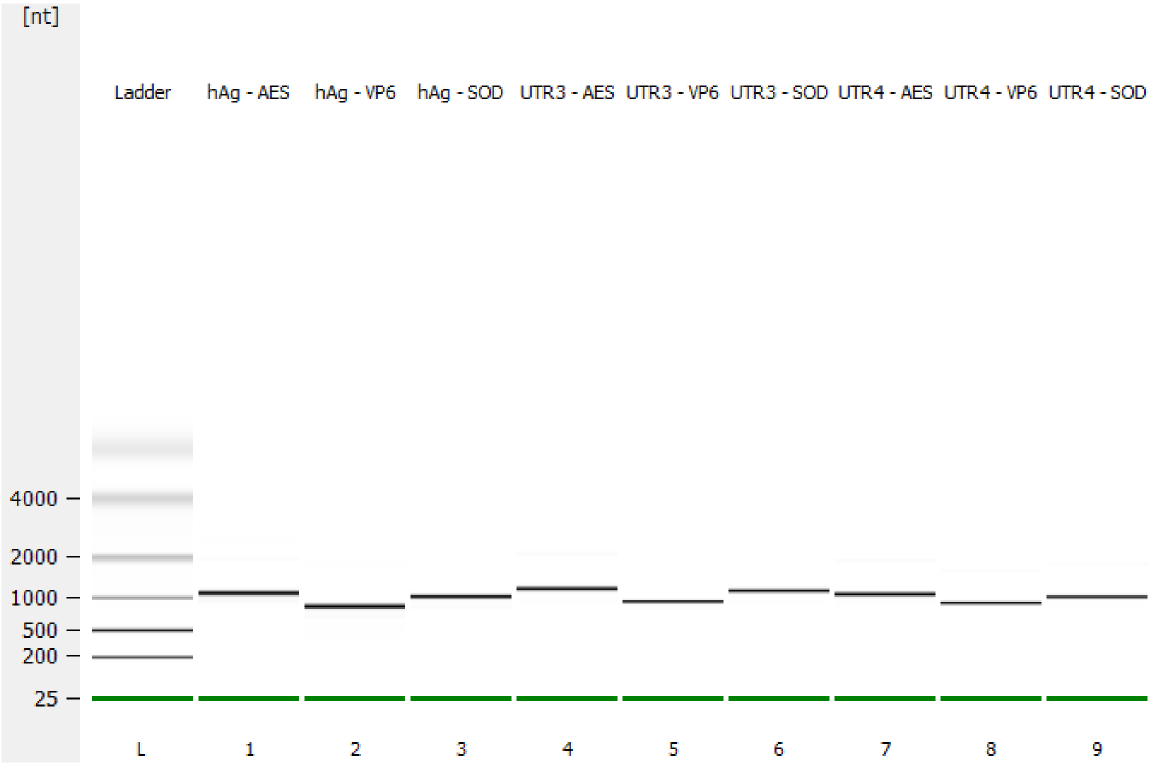

***Figure S8**: Agilent gel of IVT ARCA Nano luciferase mRNA. We confirm the good quality of our mRNAs.*

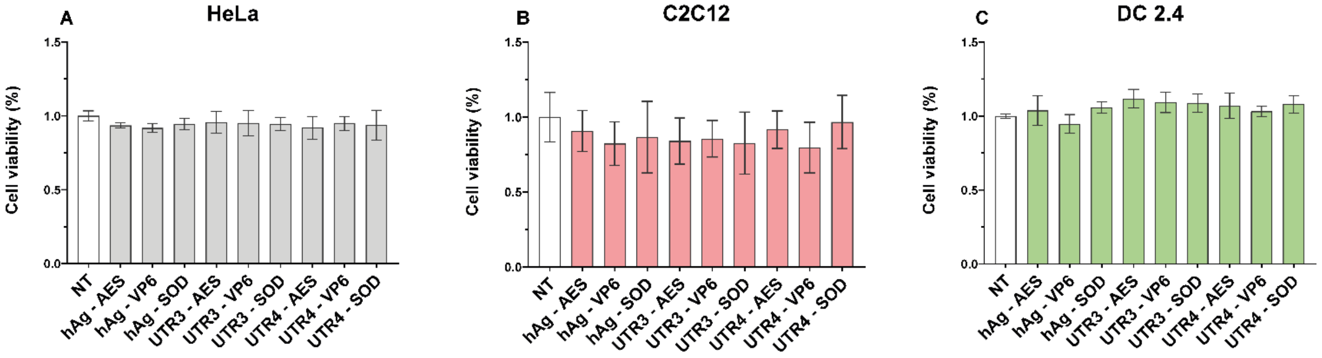

***Figure S9:** Cell viability 24 hours after transfection with Lipofectamine MessengerMAX complexes as determined by the MTT assay.*

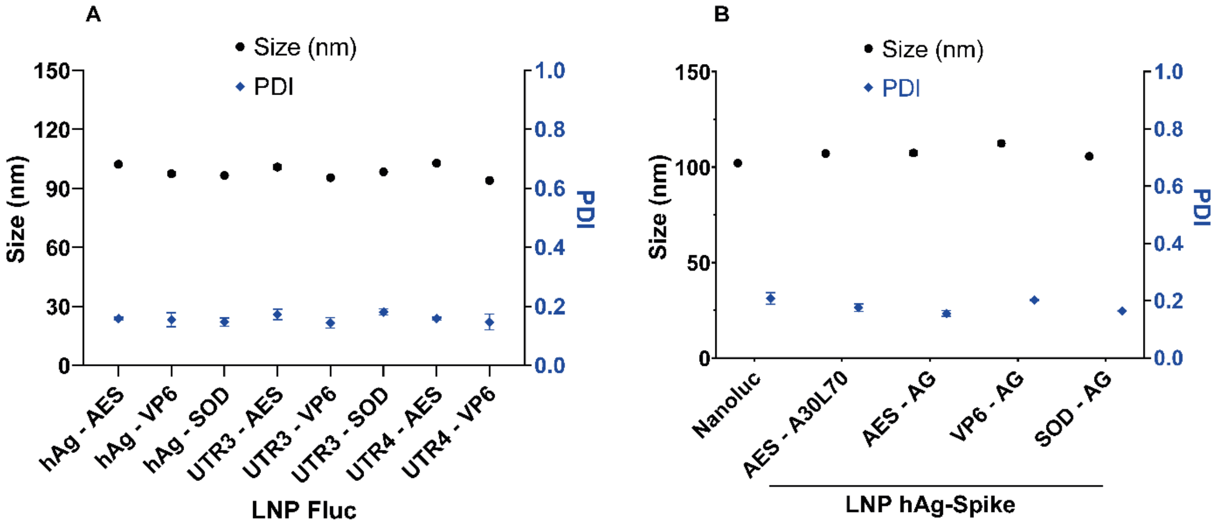

Figure S10: *LNP characterization: A) Fluc LNP, B) Spike LNP.*

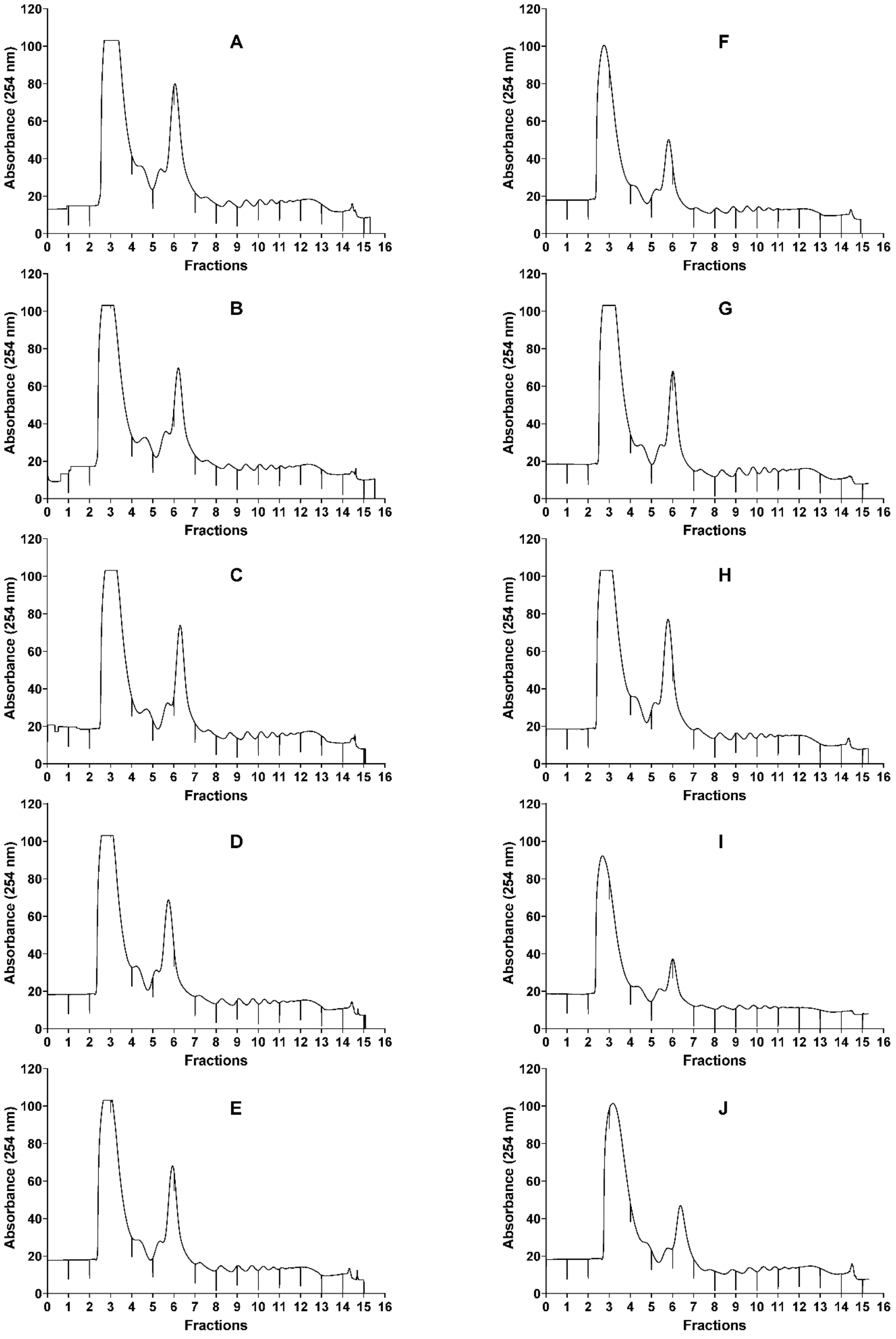

Figure S11: *Polysomes Profiles 24h after transfection: A) NT, B) hAg-AES, C) hAg-VP6, D) hAg-SOD, E) UTR3-AES, F) UTR3-VP6, G) UTR3-SOD, H) UTR4-AES, I) UTR4-VP6, J) UTR4-SOD*
