# Supplementary material for: Evaluation of synthetic mRNA with selected UTR sequences and alternative poly(A) tail, in vitro and in vivo
Source: Mol Ther Nucleic Acids. 2025 Jul 30;36(3):102648. doi: 10.1016/j.omtn.2025.102648 (PMC12355064; doi:10.1016/j.omtn.2025.102648)
Supplement: Document S2. Article plus supplemental information [file mmc2.pdf]

Molecular Therapy
Nucleic Acids
Original Article

Evaluation of synthetic mRNA with selected UTR sequences and alternative poly(A) tail, *in vitro* and *in vivo*

Ayoub Medjmedj,[1] Hugo Genon,[1] Dounia Hezili,[1] Albert Ngalle Loth,[1] Rudy Clemençon,[1] Cyril Guimpied,[1] Lucile Mollet,[1] Anne Bigot,[2] Frank Wien,[3] Josef Hamacek,[1] Clément Chapat,[4] and Federico Perche[1]

[1]Centre de Biophysique Moléculaire CNRS UPR4301, 45071 Orléans, France; [2]MyoLine, Sorbonne Université, Inserm, Institut de Myologie, Centre de Recherche en Myologie, 75013 Paris, France; [3]Synchrotron SOLEIL L'Orme des Merisiers, Saint-Aubin - BP 48, 91192 GIF-sur-YVETTE Cedex, France; [4]Centre for Integrative Biology (CBI), Molecular, Cellular and Developmental Biology (MCD), CNRS/Université de Toulouse 118 Route de Narbonne, bâtiment IBCG, 31062 Toulouse Cedex 09, France

Messenger RNA (mRNA) has emerged as an attractive new technology of drugs. The efficacy of mRNA technology depends on both the efficiency of mRNA delivery and translation. Untranslated regions (UTRs) and the poly(A) tail play a crucial role in regulating mRNA intracellular kinetics. Intending to improve the therapeutic potential of synthetic mRNA, we evaluated various UTRs and tail designs, using Pfizer-BioNTech coronavirus disease 2019 (COVID-19) vaccine sequences as a reference. First, we screened six 5′ UTRs (cap-dependent/-independent), evaluated nine 5′ UTR-3′ UTR combinations, and a novel heterologous A/G tail in cell models, and *in vivo* using luciferase as a reporter gene. Then, to decipher the translation mechanism of selected UTRs, we correlated mRNA expression with ribosome load, mRNA half-life, mRNA immunogenicity, and UTR structures. Our results showed that the heterologous tail we introduced is as potent as the Pfizer-BioNTech tail and confirmed the high potency of the human α-globin 5′ UTR. They also revealed the potential of the VP6 and SOD 3′ UTRs. We validated our results using mRNA encoding the SARS-CoV-2 spike protein formulated as lipid nanoparticles (LNPs) for mouse immunization. Overall, the selected 3′ UTRs and heterologous A/G tail have great potential as new elements for therapeutic mRNA design.

INTRODUCTION

The extraordinary success of mRNA vaccines against coronavirus disease 2019 (COVID-19) has shown the potential of mRNA for a wide range of applications.[1,2] Synthetic mRNA has the same primary structure as mature mRNA molecules, typically consisting of five elements (from 5′ to 3′): a cap structure, a 5′ untranslated region (5′ UTR), a coding sequence, a 3′ UTR, and the poly(A) tail. The production of synthetic mRNA by *in vitro* transcription (IVT) is simple, fast, and cost-effective at any scale. The starting point for IVT mRNA production is the DNA template, which can be a PCR product or linearized plasmid DNA, typically carrying a phage promoter (i.e., T7) and domains of the mRNA: 5′ UTR, ORF, 3′ UTR, and particularly the poly(A) tail. Several parameters can significantly affect the quality of IVT mRNA, including DNA template quality, reaction components, capping strategy, and downstream purification methods (reviewed by Lenk et al.)[3] The structural elements of mRNA modulate mRNA stability, translation efficiency, and overall performance. These domains are finely optimized to enhance the potency of the synthetic mRNA (reviewed by Kang et al.).[4] In the present study, we focused on UTR and poly(A) domain optimizations.

The translation initiation efficiency of mRNA is affected by the sequence and structure of the 5′ UTR region.[5] While UTRs are not translated, they contain regulatory elements that control mRNA half-life, intracellular localization, and translation rate by interacting with translation factors, mRNA-binding proteins, and microRNAs (miRNAs).[6,7] A significant amount of research explored the optimization of 5′ and 3′ UTRs using different technologies to identify sequences that can improve the translatability of mRNA.[8–12] For example, the 5′ and 3′ UTRs of the mRNA encoding the SARS-CoV-2 Spike protein in Moderna and Pfizer-BioNTech COVID-19 vaccines are different in their sequence and structure. In the Moderna mRNA vaccine, they used a 5′ UTR of undisclosed origin along with the 3′ UTR from human α-globin, while the Pfizer-BioNTech mRNA vaccine contains the 5′ UTR of human α-globin, which was identified as one of the top 20% 5′ UTRs in massive screening studies,[10,11,13] and the 3′ UTR AES-mtRNR1 combination of two genes, AES (Amino Enhancer of Split) and mtRNR1 (12S mitochondrial RNA).[12] An additional class of mRNA 5′ UTR consists in the Internal Ribosome Entry Sites (IRES), which can initiate translation in a cap-independent manner. IRES are complex RNA structures found in some viral (e.g., EMCV) and cellular mRNAs (e.g., VCIP).[14] However, predicting the optimal combination of 5′ and

Received 12 March 2025; accepted 25 July 2025;
https://doi.org/10.1016/j.omtn.2025.102648.

Correspondence: Federico Perche, Centre de Biophysique Moléculaire CNRS UPR4301, 45071 Orléans, France.
E-mail: federico.perche@cnrs.fr

© 2025 The Author(s).
Published by Elsevier Inc. on behalf of The American Society of Gene and Cell Therapy.
This is an open access article under the CC BY license (http://creativecommons.org/licenses/by/4.0/).

3′ UTR remains challenging due to tissue and cell-type dependencies, and RNA folding of UTRs, which can be affected by the coding sequence.

Another key element in mRNA translation and stability is the poly (A) tail. It protects the mRNA from degradation[15] and is required to form a closed loop between the 5′ and 3′ ends of the mRNA, a conformation that stimulates translation.[16] However, the presence of the poly(A) tail on the plasmid template for mRNA production is not stable and prone to shortening in bacteria during plasmid DNA amplification.[17] BioNTech demonstrated that poly (A) stretches of 30 and 70 adenosines separated by a 10 nt linker (A30L70 tail) resulted in more stable poly(A) tails on plasmid templates.[18] Other poly(A) tails have been developed by researchers using different strategies.[17–20]

In this study, we aimed to enhance the translatability of synthetic mRNA and establish informed structure-activity relationships of mRNA containing a selection of UTR sequences and poly(A) tails. To achieve this, we first introduced another solution to overcome the instability of poly(A) tails on plasmid templates by using a novel heterologous A/G tail design (A/G tail). We evaluated the stability of our A/G tail in *Escherichia coli* and assessed its performance for mRNA translation both *in cellulo* and *in vivo*. We then compared the translation efficiency of seven different 5′ UTRs from mammalian, viral, and synthetic origins in three different cell lines. The top three 5′ UTRs were combined with three different 3′ UTRs (AES-MT, a fragment of rotavirus gene 6 (VP6), and the human MnSOD 3′ UTR [SOD]). Using a set of nine 5′ UTR-3′ UTR combinations on a reporter mRNA, we systematically evaluated their translation efficiency, stability, immunogenicity, and ribosome load. Additionally, we characterized UTRs structures/folding by circular dichroism. To further validate the potency of selected UTRs in a vaccination context, we quantitated their vaccination potency using Spike mRNA delivered as lipid nanoparticles (LNPs) in mouse models. Our findings demonstrate the therapeutic potential of mRNAs harboring A/G tail and VP6 or SOD 3′ UTRs.

RESULTS

Alternative poly(A) tail

Stability of poly(A) tail sequences on DNA templates in bacteria

The presence of poly(A) tail in plasmid DNA templates for synthetic mRNA production is prone to truncation due to recombination events during bacterial growth. To overcome this instability, we proposed an alternative adenosine/guanosine mixed poly(A) tail (A/G tail) design using 12 repeating units of the AAAAAAAAAG motif (Table S1). Guanosine residues are the most frequent non-adenosine nucleotides in poly(A) tails in humans and other species.[20,21] Furthermore, guanylation has been reported to stabilize mRNA by reducing deadenylation[20,22] and that cellular mRNAs with non-A tails are highly translated.[22]

We compared the instability of three types of poly(A)-encoding sequences within plasmid template in bacteria: a homopolymeric tail of 120 adenosines (A120), the poly(A) tail used in the Pfizer-BioNTech vaccine consisting of two tracts of 30A and 70A joined by a 10-nucleotide linker (A30L70 tail),[18] and our novel A/G tail (Figure 1A; Table S1). We measured the length of the tail after 10 passages in bacteria by sequencing compared to the length onto the parent plasmid. We found that only 10% of the clones with A120 tail retained the full 120A tract, confirming the reported instability of homopolymeric A tails.[17] In contrast, the A30L70 and A/G tails demonstrated high stability, with 90% and 100% of clones keeping the integrity of the poly(A) tail, respectively (Figure 1B). While the reduced instability of the A30L70 tail has been demonstrated in patents and clinical use,[13,18] the A/G tail showed superior stability and appears to be a valuable alternative.

Structural properties of tail sequences

Next, we used synchrotron radiation circular dichroism (SRCD) to determine the effect of poly(A) tail selection on mRNA conformation and stability.[23,24] CD spectra of mRNA with different tail domains and transitions in secondary structure were monitored by SRCD thermal scans (15°C–95°C) (Figures 1C–1E). Positive bands at 180 and 260–265 nm and negative one between 200 and 210 nm are indicative for the constitutive A-form of mRNA.[25] Spectral profiles of mRNA with the three types of tails were very similar and indicative of A-form helices. As peaks between 260 and 270 nm are influenced by intra- and intermolecular base-pairing, that is base sequence, we monitored the SRCD changes at 260–265 nm, corresponding to different unfolding trajectories, to calculate melting temperatures (T_m) (Figure 1B). A30L70-tailed mRNA had a higher T_m (76.6 ± 1.3°C) compared to A120 (69.4°C ± 1.1°C) and A/G-tailed mRNA (69.8°C ± 1.4°C). This indicates a stabilization effect of the 10 nt linker present in A30L70.

Evaluation in cellular models

To assess the expression potential of mRNAs with different tail compositions, we generated plasmids with A30L70 or A/G tails, both encoding the NanoLuc luciferase (Nluc) reporter gene, flanked by the benchmark human α-globin 5′ UTR (hAg) and AES-mtRNR1 3′ UTR (AES), with either A30L70 or A/G tails. ARCA-capped mRNAs were synthesized from these constructs and transfected into HeLa human cancer cells to compare their translation efficiency. Kinetic analysis revealed a biphasic pattern in protein expression: during the first 24 h post-transfection, no significant difference in bioluminescence was observed between A30L70-mRNA and A/G-mRNA. However, from 24 to 96 h, A30L70-mRNA exhibited a progressively higher bioluminescence signal, reaching up to 2-fold greater intensity at 96 h (Figure 2A).

The total amount of protein produced was quantified using area under the curve (AUC) analysis, which showed that A/G-mRNA produced 25% less protein overall compared to A30L70-mRNA (Figure 2B). These results indicate that while the A/G tail demonstrated comparable efficacy to A30L70, the latter confers a slight advantage in sustaining long-term protein expression.

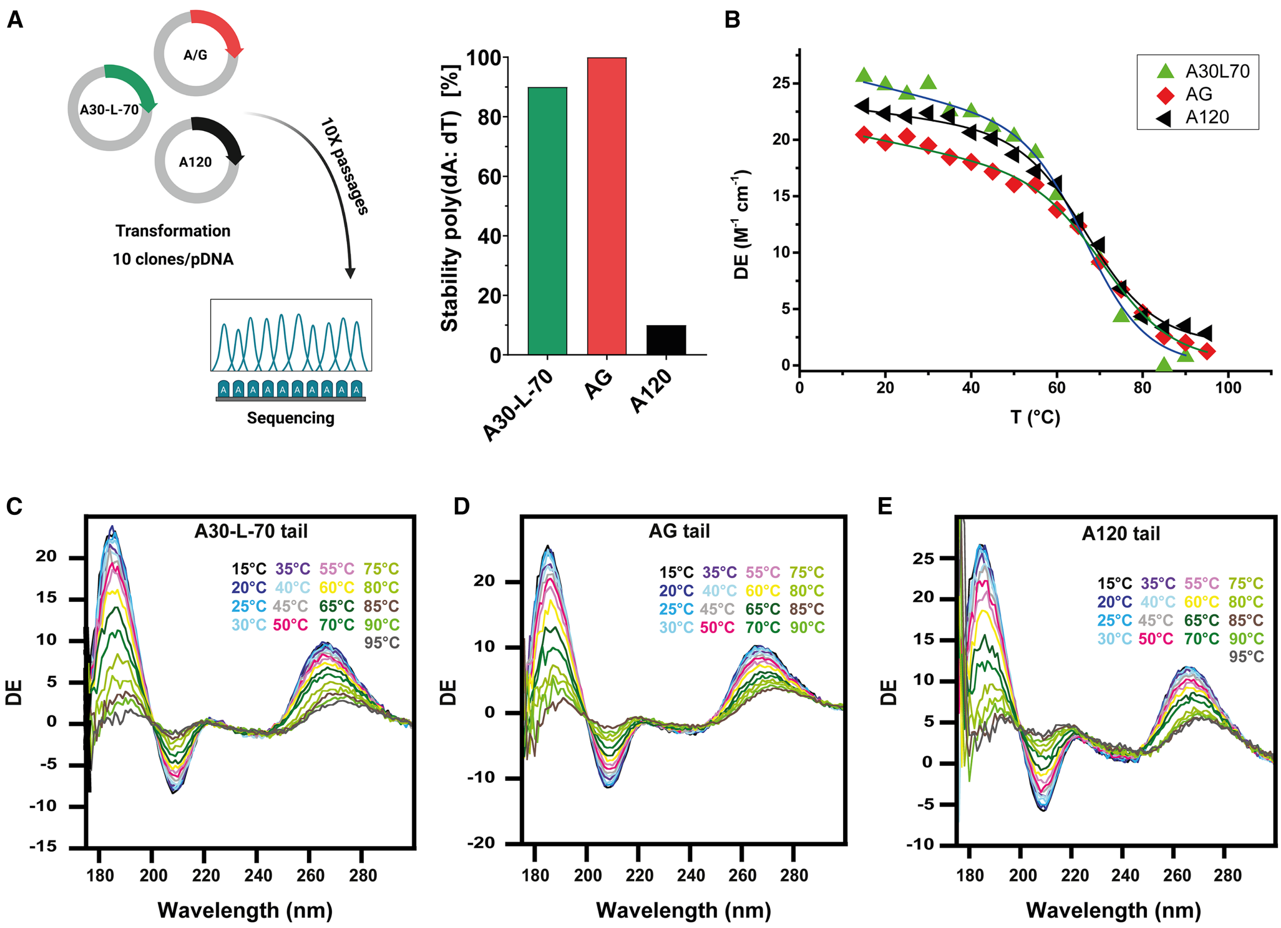

Figure 1. Stability of poly(A) tail sequences on DNA templates in bacteria and circular dichroism profiles of mRNA with the different poly(A) tails

(A) Tail stability of A30-L-70, AG, and 120A plasmids in *E. coli* DH5-α. The percentage of tail stability represents the proportion of clones maintaining tail integrity relative to the total number of clones. mRNA with different tail sequences were analyzed by synchrotron radiation circular dichroism (SRCD) by performing a thermal scan from 15°C to 90 or 95°C. (B) Melting temperatures (Tm) of mRNA tails determined by SRCD analysis. (C) SRCD scans of A30-L-70 tail sequence, (D) SRCD scans of AG tail sequence, (E) SRCD scans of A120 tail sequence. DE, Delta Epsilon.

Evaluation in vivo

We next evaluated the impact of A/G tail on mRNA translation *in vivo*. LNPs with the same composition as the Pfizer-BioNTech vaccine[23] were used to deliver A30L70- or A/G-tailed mRNA encoding firefly luciferase (Fluc) by intramuscular injection (Figures 2C and 2D). As illustrated in Figure 2C, mRNAs with both tails exhibited comparable bioluminescence expression over the 72-h period post-administration. The AUC analysis revealed that A/G tail produced only 10% less total protein compared to A30L70 (Figure 2E). These results demonstrate that the A/G tail is as potent as A30L70 and represents a viable alternative for mRNA design.

5′ UTR selection

The sequence and structure of the 5′ UTR play a crucial role in mRNA translatability, as this region serves as the primary site for ribosome recruitment and translation initiation. Many researchers have attempted to optimize this region using diverse approaches, including the selection of highly translated mRNAs and generation of de novo synthetic 5′ UTRs based on high ribosome load.[9–11] To identify the most effective 5′ UTR from previous studies, we conducted a side-by-side comparison of the translation potency of both cap-dependent and cap-independent 5′ UTRs. Our selection included the reference 5′ UTR from human α-globin (hAg) used in the BioNTech COVID-19 vaccine[10,11]; the human Hsp70 5′ UTR reported to be a general enhancer of mRNA translation[26]; the synthetic 5′ UTR UTR4 allowing strong expression of mRNA in cells and Zebrafish[27]; and the synthetic 5′ UTR neoUTR3 showing enhanced protein production (UTR3).[9] We also included the human vascular endothelial growth factor and type-1-collagen-inducible protein (VCIP) IRES[28]; the encephalomyocarditis virus (EMCV) IRES[28]; and the IRES from Cricket paralysis virus (CrPV) that is able to directly recruit the 60S subunit.[29] Each of these 5′ UTRs was cloned upstream of the Nluc reporter gene (Figure 3A), and the resulting constructs were transcribed into mRNAs with A/G tail. These mRNAs were then delivered into

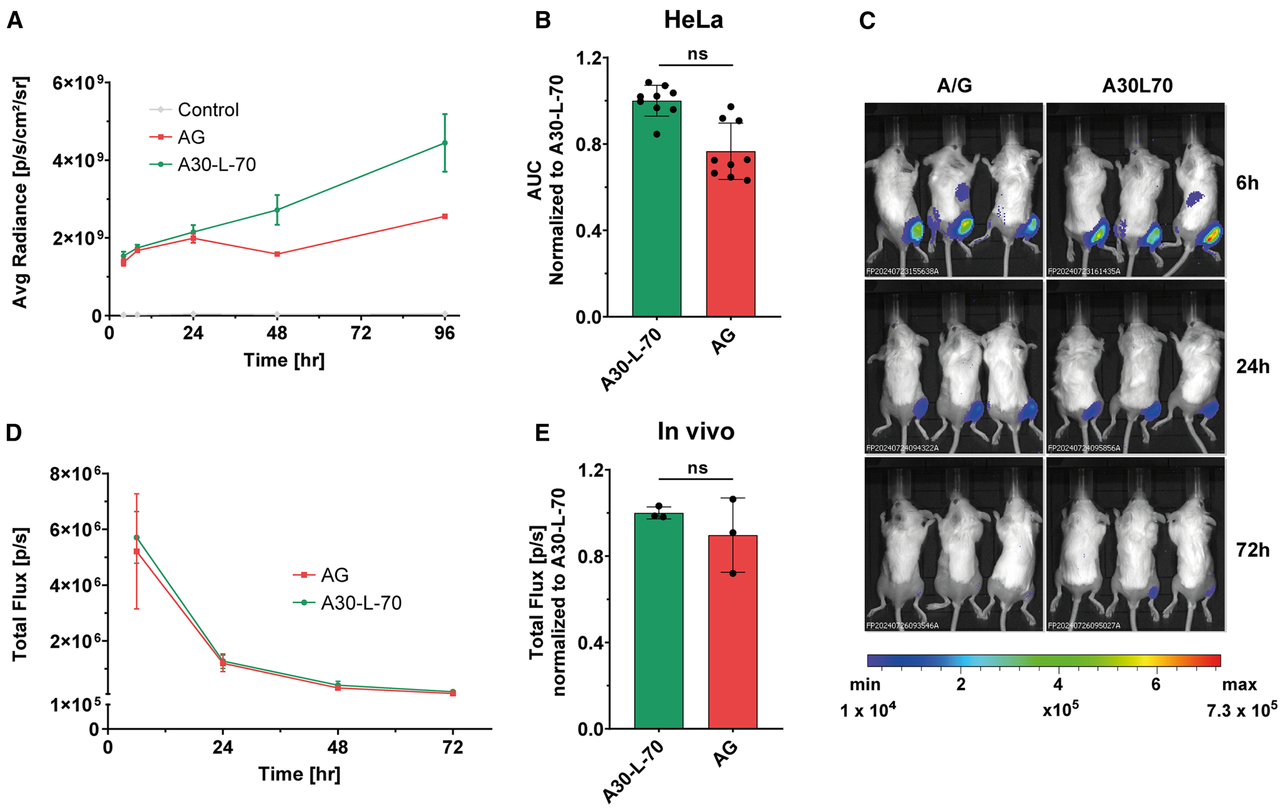

Figure 2. The potency of a novel heterologous poly A/G tail

(A) Time course analysis of nano luciferase reporter gene (Nluc) expression in HeLa cells transfected with A30-L-70 and AG ARCA-mRNAs. The measurements were acquired at 4, 8, 24, 48, and 96 h post-transfection, and results are expressed as mean ± SD. Control is untransfected cells. (B) Based on these data, total protein expression over time (the area under the curve) was calculated and normalized to A30-L-70 mRNA. Statistical significance was assessed by nested one-way ANOVA. $*p < 0.05$; $**p < 0.01$; $***p < 0.001$ ($n = 3$ independent experiments, each three technical replicates). (C) *In vivo* translation of firefly-luciferase-encoding (Fluc) mRNAs containing AG or A30-L-70 tails in BALB/c mice after i.m. injection (one experiment with three mice per group). (D) Quantification was done for all available mice per time point (6, 24, 48, and 72 h); (E) total protein expressed over time (the area under the curve) was calculated and is normalized to that of the A30-L-70 containing mRNA. One-way ANOVA, Tukey's post-test; $*p < 0.05$; $***p < 0.001$.

HeLa cells, murine myoblasts (C2C12), and murine dendritic cells (DC2.4) to evaluate their translation efficiency.

Bioluminescence was evaluated 24 h post-transfection. The results showed that the translation of mRNAs containing IRES 5′ UTRs derived from mammalian or viral sources (VCIP, CrPV, EMCV) was less than 20% of the luciferase expression achieved with the cap-dependent human alpha-globin 5′ UTR across the three cell lines tested (Figures 3B–3D). This inefficient translation of mRNAs harboring viral IRES sequences may be attributed to several factors, including the structural folding of the IRES region within the IVT mRNA and the cellular environment, as IRES elements are known to function more effectively under stress conditions.[30] Contrary to previous studies that reported higher translation efficiency of VCIP IRES compared to EMCV IRES in cell lines using bicistronic plasmid DNA,[28] our findings demonstrated similarly low translation potency for both IRES elements in the tested cell lines. Importantly, cell viability remained at or above 90% for all mRNAs, indicating good tolerability of mRNAs with these 5′ UTR sequences (data not shown).

Interestingly, the performance of the Hsp70 5′ UTR varied among the three cell lines: it exhibited lower translation efficiency than α-globin in C2C12 cells, equivalent performance in HeLa cells, and superior translation in DC 2.4 cells. The higher expression of Hsp70 5′ UTR-mRNA in DC 2.4 may be attributed to an enhancer element in its sequence and to its capability to promote translation via both cap-dependent and cap-independent mechanisms.[26,31] Furthermore, this increased expression over the mRNAs harboring viral IRES 5′ UTRs is consistent with findings from previous studies.[31] We also observed comparably high Nluc expression levels with mRNAs containing hAg, UTR3, or UTR4 5′ UTRs across all three cell lines tested. These results illustrated that cap-dependent 5′ UTRs exhibit significantly higher translation efficiency compared to cap-independent 5′ UTRs. Consequently, human alpha-globin, neoUTR3, and UTR4 5′ UTRs were selected for further investigations on 3′ UTR selection.

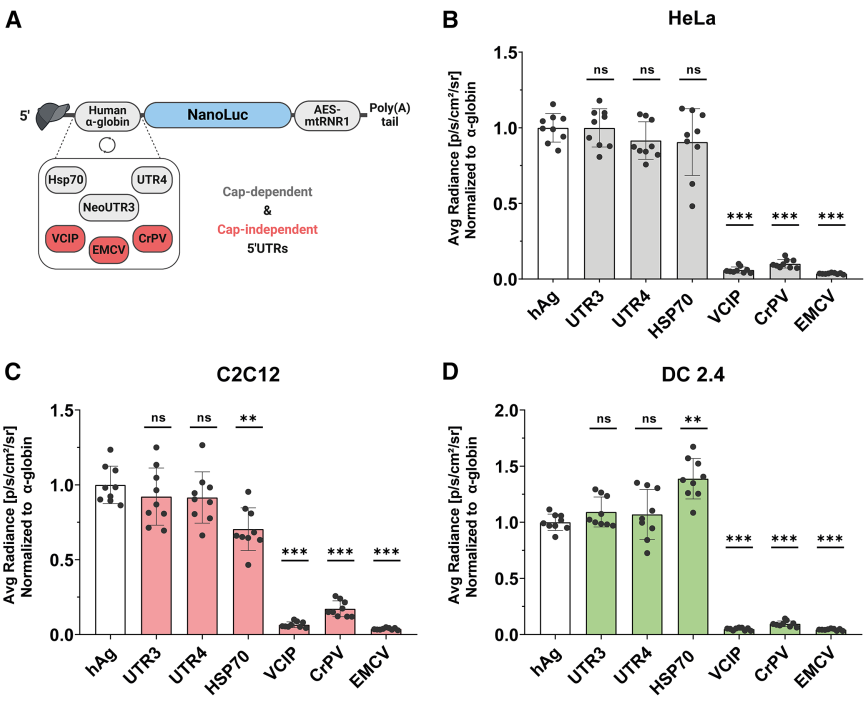

Figure 3. *In cellulo* comparison of cap-dependent and cap-independent 5′UTRs

(A) Schematic representation of 5′ UTR motifs cloned upstream of the nano luciferase reporter gene (Nluc). (B–D) After transfection of the respective mRNAs into cell lines, luciferase activity measured in relative light units (RLU) 24 h after transfection. Data are presented as the mean ± SD in HeLa (B), C2C12 (C), or DC2.4 cells (D). Statistical significance was assessed by nested one-way ANOVA. $*p < 0.05$; $**p < 0.01$; $***p < 0.001$. ($n = 3$ independent experiments, each three technical replicates).

Evaluation of 5′ UTR/3′ UTR combinations in cell models

To identify the most potent 5′ UTR-3′ UTR combination, we used three 5′ UTRs from the previous selection (hAg, UTR3, and UTR4) and three 3′ UTRs selected from the literature: AES-mtRNR1 3′ UTR (AES),[12] a fragment of rotavirus gene 6 3′ UTR (VP6),[32] and the human MnSOD 3′ UTR (SOD).[33] This resulted in a total of nine combinations (Figure 4A), including the benchmark 5′ UTR hAg/3′ UTR AES. We performed a comprehensive *in cellulo* comparison of these combinations by evaluating their translation efficiency, mRNA half-life, immunogenicity, and capacity to recruit ribosomes. Additionally, the structure of selected 5′ and 3′ UTRs was elucidated using circular dichroism, aiming to establish a functional-structural relationship (Figure S1).

Evaluation of translation in cell lines

First, we monitored the kinetics of mRNA translation of the nine UTR combinations in HeLa cells, C2C12 cells, and DC 2.4 cells, by measuring bioluminescence at different time points (4–96h) post-transfection; total protein production over time was determined by calculating the area under the curve (AUC). As shown in Figures 4B and 4C, eight of the nine combinations tested had similar translation kinetics and total protein production over time in HeLa cells. The UTR4-SOD mRNA was the only mRNA that produced less protein than the benchmark. Noteworthy, in C2C12 cells, hAg-VP6 and hAg-SOD combinations produced significantly a higher amount of protein than the benchmark (Figure 4D). Oppositely, the other mRNAs showed the same pattern as in HeLa cells (Figure 4C). Remarkably, in DC 2.4, we found that the UTR4-SOD mRNA pair was translated 2-fold less than the benchmark, while the other mRNAs showed similar protein expression (Figure 4E).

Vaccination is the only approved application of synthetic mRNA.[2,4] COVID-19 vaccines required intramuscular administration of mRNA-LNP and expression of the mRNA-encoded antigen in antigen-presenting cells such as dendritic cells (DC) to induce a protective immune response.[34] Accordingly, we evaluated the nine UTR combinations in functionally relevant AB1079-immortalized human myoblasts (Figure 4F) and human monocyte-derived DCs (MoDCs) (Figure 4G). The results in AB1079 cells mirrored the pattern obtained in C2C12 cells, except for UTR3-SOD, UTR4-VP6, which produced 25% less protein, and UTR4-SOD mRNA, which produced less than 50% of the protein compared to the benchmark mRNA (Figure 4F). In MoDCs, five of the nine mRNAs tested were similarly translated (hAg-AES, hAg-VP6, hAg-SOD, UTR3-AES, and UTR4-AES), and mRNA with 3′ UTR VP6 or SOD in combination with UTR3 or UTR4 5′ UTRs resulted in decreased mRNA translation (Figure 4G). A possible explanation for the lower expression of UTR4-SOD mRNA could be a difference in interferon pathway activation, resulting in decreased mRNA translation.[35] However, all the constructs had the same percentage of dsRNA content (Table S2) and induced comparable activation of the interferon pathway in DC2.4 cells (Figure S2). Furthermore, in cell-free translation assays, the nine mRNAs were similarly translated (Figure S3A), suggesting that the differences observed in the different cell lines may be due to the cell environment/context/specificities and/or competition with endogenous mRNAs.[36] Moreover, the translation of all constructs except CrPV was inhibited by 90% using the canonical eIF4G initiation pathway inhibitor (4EGI-1).

As the choice of cap analog influences the initiation of translation, we also evaluated the translatability of three mRNAs harboring the CleanCap AG trinucleotide cap analog used in the Pfizer-BioNTech vaccine.[37,38] We compared the translation efficiency of ARCA- and CleanCap-AG-capped mRNA with alpha-globin as 5′ UTR in DC2.4 cells and MoDCs. The results showed similar patterns for

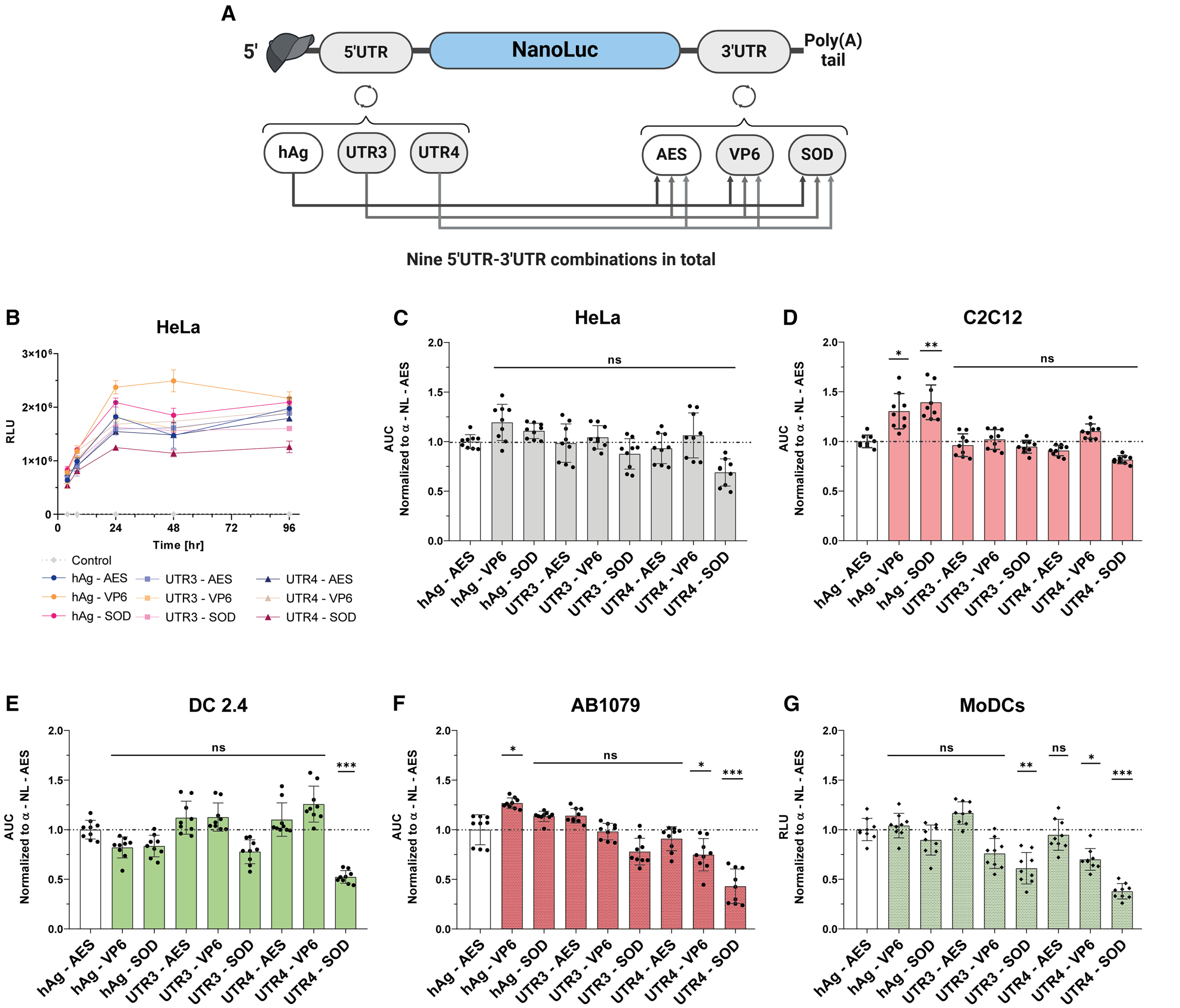

Figure 4. Evaluation of 5′ and 3′UTR combinations in cell models

(A) Schematic representation of nine constructs generated by cloning different 5′ UTR motifs upstream and 3′ UTR motifs downstream of the nano luciferase reporter gene (Nluc). (B) Time course analysis of Nluc expression in HeLa cells transfected with the nine mRNA constructs. Bioluminescence was acquired at 4, 8, 24, 48, and 96 h post-transfection, and results are expressed as mean ± SD. (C–F) Total protein expression over time (area under the curve) calculated from time course data and normalized to the hAg 5′UTR-AES 3′UTR containing mRNA in HeLa cells (C), or murine myoblasts (C2C12) (D), or murine dendritic cells (DC2.4) (E), or human myoblasts (AB1079) (F). Statistical significance was assessed by nested one-way ANOVA. *$p < 0.05$; **$p < 0.01$; ***$p < 0.001$. (three independent experiments with three technical replicates). (G) The nine mRNAs were transfected to monocyte-derived dendritic cells (MoDCs). Relative light units (RLU) were measured 24 h post-transfection. Data are presented as mean ± SD. Statistical significance was assessed by nested one-way ANOVA. *$p < 0.05$; **$p < 0.01$; ***$p < 0.001$. (n = 3 independent donors, each three technical replicates).

the three mRNAs regardless the cap analog. However, as expected, mRNAs with CleanCap AG yielded two times more protein than mRNAs with the ARCA cap analog (Figure S4). Altogether, these results prove that the expression is cell-type dependent, relieving the difficulty in predicting the efficacy of UTR combinations and reinforcing the need for evaluation in cellular and *in vivo* models.

Half-life and polysome profiling of mRNAs with UTR combinations

To understand the difference of protein production observed previously, we analyzed the most likely reasons for differences in translation efficiency: the mRNA half-life, ribosomes loading, and UTR structures.[12,39]

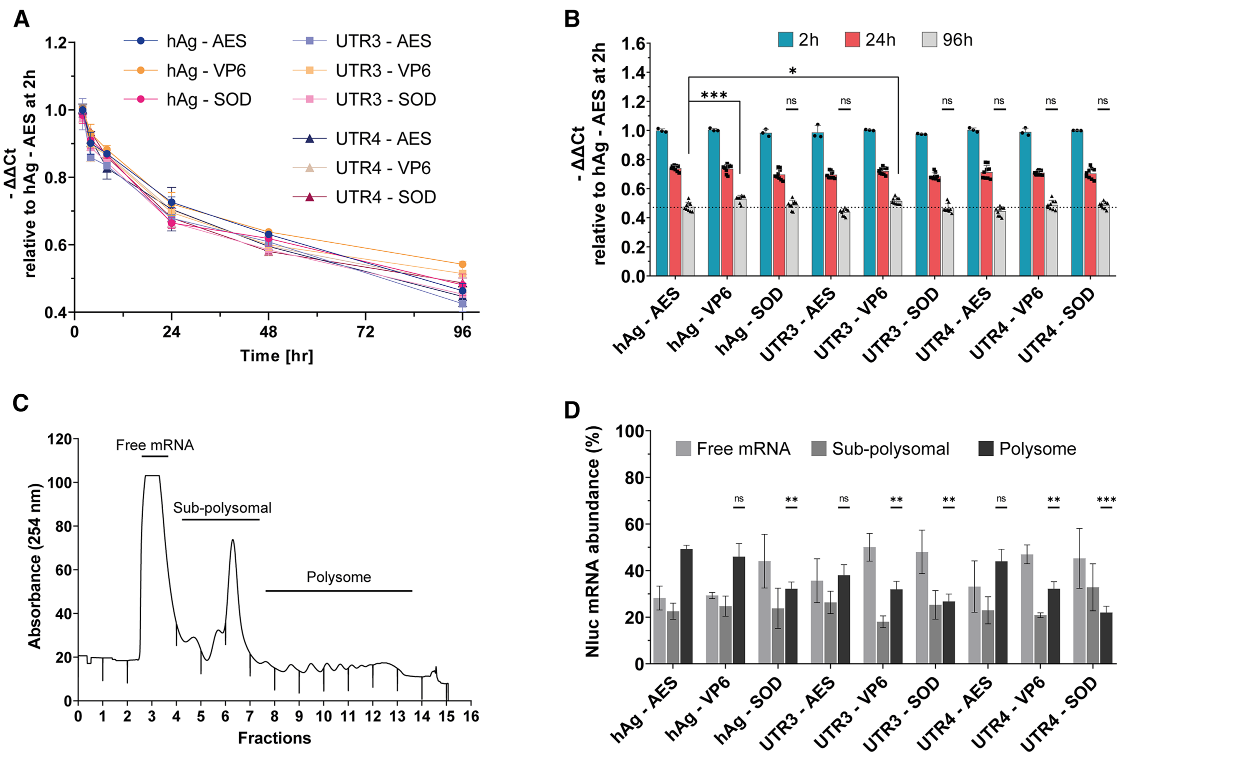

Figure 5. mRNA stability and ribosome load capacity

(A) Average stability of the mRNA was assessed by RT-qPCR harvested at the indicated time points. The transcript levels were determined via ΔΔCT calculation relative to hAg-AES mRNA and are shown relative to the 2 h. (B) Nluc mRNA levels at 2, 24, and 96 h relative to 5′hAg-3′AES at 2 h Data are presented as mean ± SD. Statistical significance for 96 h was assessed by nested one-way ANOVA. *$p < 0.05$; **$p < 0.01$; ***$p < 0.001$. (C) Polysome profile from HeLa cells 24 h after transfection, analyzed on a 10%–50% sucrose gradient. (D) Nluc mRNA levels assessed by RT-qPCR from each polysome fraction and presented in three groups: free mRNA, sub-polysomal-associated mRNA, and polysome-associated mRNA. Results are expressed as mean ± SD of two independent experiments with three technical replicates. Statistical significance for polysome fraction was assessed by one-way ANOVA, Dunnett's post-test; *$p < 0.05$, ***$p < 0.001$.

mRNA stability

To assess the stability of the 9 mRNAs, HeLa cells were transfected with Nluc mRNAs, and RT-qPCR was performed at different time points (2–96 h) to measure their half-life. All tested mRNAs exhibited a similar time-dependent decay and a comparable half-life, except for mRNA hAg-VP6, which seemed to be slightly more stable than the benchmark mRNA, especially after 96 h (Figures 5A and 5B). We completed mRNA half-life measurements with a comparison of mRNA content of cells treated with mRNA harboring different 5′ UTR α-globin combinations at the single-cell level using FISH-Flow.[40] FISH-Flow results showed comparable Nluc mRNA levels at 2 and 24 h after transfection for hAg-AES, hAg-VP6, and hAg-SOD mRNAs (Figure S5A). These results supported similar kinetics decay for the nine mRNA transcripts, indicating that the difference observed in translation potency is not caused by mRNA half-life.

Polysome profiling

Increased translation efficiency of a particular UTR combination would be expected to result in its accumulation in polysomal fractions. To investigate this, HeLa cells were transfected with IVT Nluc mRNA harboring the nine UTR combinations and harvested after 24 h. Cytosolic extracts were then fractionated on 10% to 50% sucrose gradient, and RT-qPCR was performed on each fraction. All HeLa cells transfected with different mRNAs exhibited similar sub-polysomal (40S, 60S, and 80S) and polysomal profiles (Figure 5C). However, the distribution of IVT Nluc mRNA within polysome fractions varied among the UTR combinations. Notably, hAg-AES, hAg-VP6, and UTR4-AES showed more pronounced association with polysomes, while UTR3-SOD and UTR4-SOD showed lower association (less than 25%) (Figure 5D). Interestingly, these two mRNAs (UTR3-SOD and UTR4-SOD) with reduced polysomal abundance also yielded less protein than the benchmark mRNA (Figure 4). This polysome profiling analysis revealed that the efficiency of mRNA translation depends on the specific combination of 5′ UTR and 3′ UTR sequences, rather than only on the 5′ UTR sequence.

The SRCD with thermal scans (15°C–95°C) was used to compare the structure and Tm of each UTR. 5′ UTRs and 3′ UTRs exhibited very similar CD spectra (Figure S1). However, UTR4 had a drastically lower T_m (59.6°C ± 1.6°C) compared to hAg (79.2°C ± 0.9°C) and UTR3 (76.3°C ± 0.9°C). T_m of the 3′ UTRs were

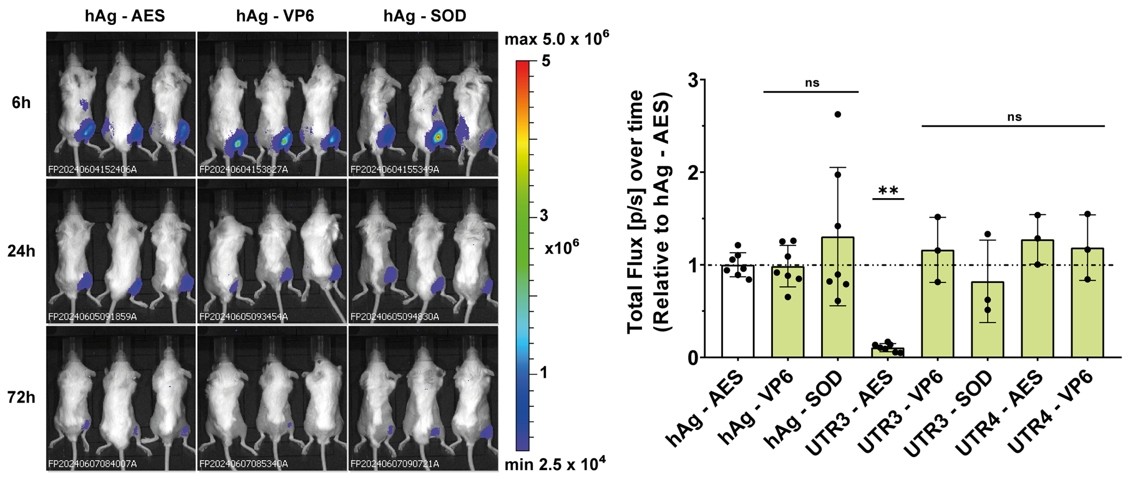

Figure 6. Evaluation of 5′ and 3′UTR combinations in mice

Bioluminescence imaging of firefly luciferase (FLuc) expression in BALB/c mice following intramuscular (i.m.) injection of mRNA constructs. Representative images of three mice per group are shown at 6, 24, and 72 h post-injection. Quantification was done for all available mice per time point (6, 24, 48, and 72 h). Total protein expressed over time (the area under the curve) was calculated and is normalized to that of the hAg 5′UTR- AES 3′UTR containing mRNA. One-way ANOVA, Tukey's post-test; *$p < 0.05$, ***$p < 0.001$ (one experiment with three to seven mice as indicated).

equivalent: 76.6°C ± 1.4°C for AES-mtRNR1, 76.4°C ± 0.7°C for VP6, and 69.7°C ± 5.7°C for SOD.

Taken together, these results suggest that the observed differences in total protein production among mRNAs UTR combinations can be attributed to ribosome loading rather than to mRNA stability or immunogenicity.

Evaluation of 5′ UTR/3′ UTR combinations *in vivo*

To assess the suitability of UTR combinations for therapeutic applications, eight UTR combinations were evaluated *in vivo* in mouse models using firefly luciferase as a reporter gene. The combination 5′ UTR4-3′ SOD was excluded from the study due to its low translation efficiency especially in MoDCs (Figure 4G). BALB/c mice received 1 μg of mRNA LNPs via intramuscular injection (Figure 6). Luciferase activity was imaged at different time points, and total protein production was calculated after 72 h. Total protein production reached equivalent levels for all mRNAs except for UTR3-AES mRNA, which produced 10-fold less protein than hAg-AES mRNA (Figure 6). The poor translation of the UTR3-AES combination *in vivo* was not expected, as it had performed well in cell lines with nanoluciferase. In line with the *in vivo* findings, the transfection of HeLa cells with Fluc mRNA containing UTR3-AES resulted in lower protein production than the benchmark mRNA (hAg-AES) (Figure S6). To investigate this unexpected poor performance of UTR3-SOD Fluc mRNA, we conducted an *in vitro* translation assay using preheated and unheated mRNA. Interestingly, the preheated UTR3-AES Fluc mRNA produced two times more protein than the unheated mRNA, whereas UTR3-VP6 Fluc mRNA produced the same amount of protein regardless of preheating (Figure S3B). Additionally, SRCD analysis showed that UTR3-AES Fluc mRNA had a different profile compared to UTR3-VP6 Fluc mRNA, suggesting that UTR3-AES Fluc mRNA is structured and potentially less accessible to the ribosome (Figure S7). These results indicate that the sequence of the coding region may interfere with the folding of the 5′ UTR and 3′ UTR, leading to a conformation of the mRNA less favorable for translation.

Based on the previous results, three combinations featuring the human alpha-globin as the 5′ UTR were selected for validation using mRNA that encodes the SARS-CoV-2 spike protein in a vaccination context.

Evaluation of therapeutic potential

After conducting a comprehensive study of the 5′ and 3′ UTR combinations in various cell models and *in vivo* using reporter genes (nano and firefly luciferases), we selected the three UTR combinations with human alpha-globin as 5′ UTR (hAg-AES, hAg-VP6 and hAg-SOD) along with the A/G tail. To assess the potential utility of these selected UTRs in the context of vaccine application against SARS-CoV-2, we replaced Nluc with the optimized spike protein sequence used in the Comirnaty mRNA vaccine.

First, we compared modified (m1Ψ) with unmodified Spike mRNA in HeLa cells. The results showed that the modified mRNA yielded 10 times more than the unmodified mRNA (Figure S5B). We then evaluated the three m1Ψ mRNAs in HeLa cells and AB1079 myoblast cells. The VP6 and MnSOD 3′ UTRs produced more spike protein than the AES-mtRNR1 3′ UTR (Figures 7B and 7C), which is consistent with previous results using the Nluc reporter protein (Figures 4C and 4F).

Finally, we administered LNP-formulated spike-encoding mRNA intramuscularly (i.m.) to BALB/c mice ($n = 5$). Mice were

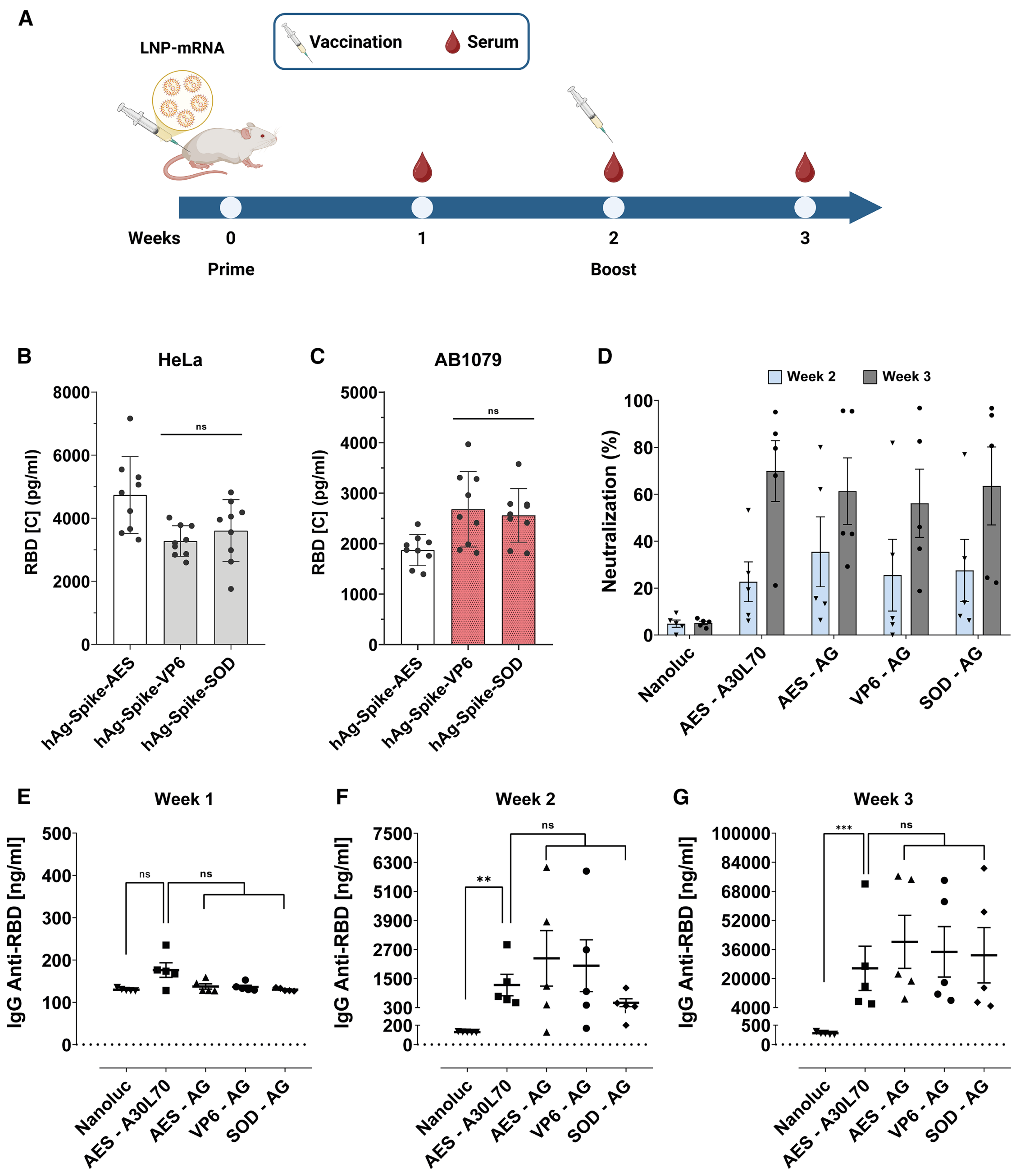

Figure 7. Spike-encoded mRNA and investigating vaccine immunogenicity in mice

(A) Schedule of vaccination and serum collection. (B and C) After transfection of the modified (m1Ψ)-spike mRNA into cells, the spike protein was measured using ELISA 24 h post-transfection. Data are presented as the mean ± SD for HeLa cells (B) and AB1079 cells (C). Statistical significance was assessed by nested one-way ANOVA. *$p < 0.05$; **$p < 0.01$; ***$p < 0.001$. (three independent experiments with three technical replicates). BALB/c mice were immunized twice with 5 μg of spike-encoded modified (m1Ψ) mRNA-LNP at weeks 0 and 2. Mice in the mock group received nanoluciferase mRNA-LNP. Spike-protein-specific IgG titers were measured at weeks 1, 2, and 3 (E–G), while neutralizing antibodies were assessed at weeks 2 and 3 (D) using ELISA ($n = 5$). Statistical significance was assessed by Kruskal-Wallis test. *$p < 0.05$; **$p < 0.01$; ***$p < 0.001$ (one experiment with five mice per group). AES, AES-mtRNR1.

immunized twice (on days 0 and 14) with LNP-formulated spike mRNA containing hAg-AES, hAg-VP6, or hAg-SOD UTR combinations with A/G tail and the benchmark Comirnaty (hAg-AES with A30L70 tail). Antibody responses against spike (total immunoglobulin G [IgG] and neutralizing antibodies) were measured by ELISA at different time points (Figure 7A). Two weeks after the first

injection, mRNA with AES-mtRNR1 or VP6 3′ UTRs induced higher production of IgG anti-RBD spike protein compared to the SOD 3′ UTR mRNA (Figure 7F). Interestingly, 1 week after the second injection (boost), no significant differences in IgG production were observed among the three 3′ UTRs (Figure 7G). Regarding the tails, the A/G tail revealed a tendency of higher IgG production after 2 and 3 weeks compared to the A30L70 tail, although the difference was not statistically significant (Figures 7F and 7G). While there were some variations in IgG production, neutralizing antibody responses were similar across groups, with a marked increase following the booster injection (Figure 7D). Altogether, these results demonstrate the potential of VP6 and SOD 3′ UTRs, as well as the A/G tail, as viable alternatives to AES-mtRNR1 and A30L70, respectively, in the context of mRNA vaccine development.

DISCUSSION

Translation efficiency and mRNA stability of synthetic mRNA are directly linked to its design elements (Cap, UTR, codon usage, and poly(A) tail), IVT parameters, and downstream purification. Optimizing these elements presents a significant challenge in developing effective mRNA therapeutics. Our study aimed to enhance the potency of synthetic mRNA and establish an informed structure-activity relationship by systematically evaluating different 5′/3′ UTR combinations and a novel poly(A) tail.

The instability of the traditional poly(A) tail during plasmid amplification in bacterial systems presents a significant challenge in mRNA production. This instability, characterized by shortening of the poly(A) sequence, directly affects the translation efficiency of the mRNA.[17] To address this issue, researchers have explored different approaches, such as including an internal 10-nucleotide linker into the poly(A) stretch.[18] In our study, we proposed a novel solution consisting of a heterologous poly(A/G) tail. This A/G tail demonstrated superior stability compared to BioNTech's A30L70 tail in bacterial systems while maintaining comparable expression levels of reporter gene *in cellulo* and *in vivo*. Moreover, it yielded similar results in vaccination against SARS-CoV-2. The structural analysis revealed that the A30L70 and A/G tails exhibited a similar T_m and SRCD profiles, suggesting comparable structural properties. The enhanced performance of the A/G tail may be attributed to increased mRNA stability within cells. Previous studies have shown that the incorporation of non-A nucleotides, particularly guanosine, can interfere with the deadenylation process, thereby enhancing mRNA stability.[41] A recent study by Liu et al. reported that mRNA tails containing C ang G residues were more efficiently translated than mRNA with pure A tails in HeLa cells.[22] This study did not evaluate stability of templates nor translation efficiency in human primary cells or *in vivo*. Our A/G tail can be further improved by optimizing the percentage of G residues and overall tail length for improved performance. Moreover, future studies will evaluate the binding of poly(A)-binding proteins (PABPs).

Another key element in mRNA translation efficiency and stability is the 5′ and 3′ UTRs. Unlike previous studies that focused on separate optimization of 5′ or 3′ UTR, we conducted a comprehensive side-by-side comparison of various combinations of 5′ and 3′ UTR derived from mammalian, synthetic, and viral origins. Our results demonstrated that cap-independent 5′ UTRs exhibited a poor translation rate across three different cell lines compared to cap-dependent 5′ UTRs, which is consistent with previous studies showing that IRES are more efficient under stress conditions.[42,43]

The evaluation of the combinations of three cap-dependent 5′ UTRs (hAg, UTR3, and UTR4) with three 3′ UTR sequences (AES, VP6, and SOD) in various cellular models revealed that improved protein production was due to increased protein translation efficiency rather than enhanced mRNA stability (Figures 4 and 5). The interactions between 5′ and 3′ UTR are crucial and can modulate the mRNA translation efficiency.[44] Our result showed that the 5′ UTR hAg yielded similar results regardless the 3′ UTR sequence across different cell models, suggesting that the 5′ UTR is the primary driver for protein translation from synthetic administered mRNA. However, the synthetic 5′ UTR UTR4 yielded different results depending on the paired 3′ UTRs (Figure 4), especially in MoDCs. Despite the 5′ UTR UTR4 being identified in high ribosome load analysis,[27] when paired with 3′ UTR SOD, it yielded 2-fold less protein compared to the reference (Figure 4). This observation is supported by the polysome profiling results (Figure 5), suggesting that ribosome load on mRNA is influenced by the 5′ and 3′ UTR base-pairing interactions.

The *in vivo* evaluation of these 5′ and 3′ UTR combinations highlighted the fact that open reading frame (ORF) may affect the mRNA secondary structure and, consequently, impact its translation efficiency.[45] The 5′ UTR neoUTR3 paired with the 3′ UTR AES-mtRNR1 yielded different results depending on the ORF used. With nanoluciferase ORF, protein levels were similar to the benchmark, whereas with firefly luciferase ORF, they were 10-fold lower compared to the benchmark *in vivo*. A possible explanation is the interaction between the UTRs and the Fluc ORF, leading to mRNA folding that is not suitable for efficient translation.[46] We confirmed this hypothesis through *in vitro* translation assays and SRCD analysis (Figures S3B and S6).

We validated the identified 3′ UTR motifs (VP6 and SOD) paired with hAg in vaccination study using SARS-CoV-2 spike protein-encoding mRNA delivered as LNPs. By selecting the 3′ UTRs, we improved protein yields by 40% compared to the benchmark in human muscle cells (Figure 7). However, *in vivo* immunization tests showed comparable levels of antigen-specific IgG production and neutralizing antibodies across the groups (Figure 7).

In addition to optimizing synthetic mRNA sequences, the parameters of the IVT and the choice of downstream purification are crucial for improved translation efficiency. We demonstrated that purification using oligo dT magnetic beads reduced but did not completely eliminate double-stranded RNA (dsRNA) contamination (Table S3). Interestingly, the use of m1Ψ led to decreased dsRNA levels, as did

the use of CleanCap AG compared to ARCA cap analog (Table S3). CleanCap AG yielded up to 2-fold more expression compared to ARCA in DC2.4 and MoDCs (Figure S4), likely due to higher capping efficiency (up to 98%) and lower dsRNA levels.

The rational approach used in our study to compare poly(A) tails and UTR sequences has broad applications for examining the effects of mRNA sequence engineering on translation. In contrast to previous studies that optimize mRNA domains separately, our study highlights the importance of considering the entire mRNA sequence, the production parameters of IVT mRNA, and the target cell types of the desired application, as factors regulating translation and mRNA stability are cell-type-specific.

We believe that the presented results may be valuable for developing a new tool for mRNA-based therapeutics optimization based on high ribosome load that considers the full mRNA sequence and intracellular environment for a precise optimization of therapeutics mRNA.

MATERIALS AND METHODS

DNA templates preparation

The plasmid pFR_CrPV_xb containing the CrPV 5′ UTR was a gift from Phil Sharp (Addgene plasmid # 11509).[47] The R5 EMCV plasmid was a gift from Vincent Mauro (Addgene plasmid # 51733).[48] UTR sequences from neoUTR3,[9] MnSOD,[33] VP6,[32] hsp70,[26] and poly(A) tails were synthesized by GenScript (Ryswick, the Netherlands). The SARS-CoV-2 spike glycoprotein sequence based on BNT162b1 was synthesized by GenScript. pF4AgNanoLuc (ATG-42) was a gift from Lance Encell (Addgene plasmid # 137777).[49]

To generate the plasmids used in this study, UTR sequences and coding sequences (CDS) for firefly luciferase (Fluc) or SARS-CoV-2 Spike protein were cloned into the pF4Ag vector. The constructs were verified by restriction enzyme analysis and Sanger sequencing performed by Eurofins (Ebersberg, Germany). The generated plasmid DNA (pDNA) was amplified in *E. coli* DH5α and purified using a NucleoBond Xtra Midi Plus Kit (Macherey Nagel) according to the manufacturer's instructions.

In vitro transcription of mRNA

For IVT mRNA production, plasmids were linearized and purified from gel using the NucleoSpinGel and PCR Clean-up Kit (Macherey-Nagel). Capped-mRNA with Anti-Reverse Cap Analog (ARCA) or CleanCap Reagent AG (Trilink) was produced by *in vitro* transcription using the HighYield T7 RNA Synthesis Kit from Jena Biosciences according to the manufacturer's protocol. The resulting IVT mRNA was purified using the Monarch RNA Cleanup Kit (NEB). Depending on experiments, additional purification with Oligo d(T)25 Magnetic Beads (NEB) was performed as described previously.[50] The integrity and quality of the purified mRNA was verified by capillary electrophoresis using an Agilent 2100 Bioanalyzer. Double-stranded RNA (dsRNA) contamination was quantified using a dsRNA ELISA Kit (Nordic Mubio) following the manufacturer's instructions. Results of mRNA quality analysis and dsRNA contamination levels are presented in Figure S8 and Tables S2 and S3.

Stability of Poly(A) tails in *E. coli*

E. coli clones carrying plasmid DNA (pDNA) with A120, A30L70, or A/G tails were passaged 10 successive times in LB broth containing ampicillin selection antibiotic (Merck). After passage 10, bacterial suspensions were harvested, and pDNA was purified using a NucleoSpin Plasmid Kit (Macherey Nagel). Integrity of tails was verified by Sanger sequencing performed by Eurofins (Ebersberg, Germany).

Synchrotron radiation circular dichroism

We followed the protocol described in Wien et al.[25] to record SRCD spectra of mRNA. Spectral magnitudes were calibrated using a solution of camphorsulfonic acid (CSA) at a known concentration. mRNA was diluted in HEPES buffer pH 7.5, 2.5 mM $MgSO_4$ at a final concentration of 1–5 mg/mL. CD spectra were recorded in CaF_2 cuvettes with a 65 μm path and an acquisition window between 170 and 320 at a 1 nm bandwidth. The buffer spectrum was subtracted from the sample spectra. We performed thermal scans from 15°C to 95°C (every 5°C or 8°C). Spectra were analyzed using CDToolX: Version 2.1.[51] Data were expressed as delta epsilon (DE) and normalized to sample concentration. We used Origin for curve fitting.

Cell culture

Cell lines

C2C12 murine myoblasts and HeLa human ovarian carcinoma cells were purchased from the American Type Culture Center. C2C12 cells were cultured in Dulbecco's modified eagle media (DMEM, Sigma) containing 10% heat-inactivated fetal bovine serum (FBS, Sigma) and 1% antibiotics (Sigma). HeLa cells were cultured in Minimum Eagle Media containing 10% FBS and 1% antibiotics. AB1079 are immortalized human myoblasts isolated from a 38-year-old healthy individual, as described in Mamchaoui et al.[52] AB1079 were cultured in skeletal cell growth medium containing 50 μg/mL fetuin, 10 ng/mL recombinant human epidermal growth factor, 1 ng/mL recombinant human basic fibroblast growth factor, 10 μg/mL recombinant human insulin, and 0.4 μg/mL dexamethasone (PromoCell). DC2.4 murine DC cells were a gift from Kenneth L. Rock.[53] DC2.4 cells were grown in RPMI 1640 medium supplemented with 10% heat-inactivated FBS. All cells were cultured at 37°C in a humidified atmosphere containing 5% CO_2.

Monocyte-derived dendritic cells

Venus blood samples were purchased from Etablissement Français du Sang (Maison du don-EFS Orléans). Peripheral blood mononuclear cells (PBMCs) were isolated using Ficoll density gradient centrifugation (11753219, Fisher Scientific) at 2,000 rpm for 20 min. Monocytes were then isolated using Dynabeads Untouched Human Monocytes Kit following manufacturer's protocol. Monocytes were differentiated into MoDC by cultivating 5 days in RPMI medium supplemented with 10% heat-inactivated FBS, human

interleukin-4 (IL-4) (62.5 ng/mL), and human granulocyte macrophage colony-stimulating factor (75 ng/mL).

mRNA transfection

All cell lines were confirmed mycoplasma-free using the MycoAlert Mycoplasma Detection Kit (Lonza). One day before transfection, cells were seeded in 96-well plates at densities of 10,000 cells per well for HeLa, AB1079, and C2C12 lines and 20,000 cells per well for DC2.4 and MoDCs. The transfection was performed with Lipofectamine MessengerMAX (LFM, Thermo Fisher) following the manufacturer's recommendations, with 100 ng of capped-mRNA per 10,000 cells. Transfection with LFM did not induce detectable cytotoxicity (Figure S9).

LNP preparation

LNPs were formulated using the same lipid composition as the Pfizer-BioNTech COVID-19 vaccine,[38] consisting of ALC 0315, DSPC, cholesterol, and ALC-0159 with a molar ratio of 50:10:38.5:1.5 and a nitrogen-to-phosphate ratio of 6. mRNA and lipid solution were mixed at a 1:3 volume ratio using the Fluigent microfluidic system (Fluigent, France) equipped with a Herringbone Mixer chip Fluidic 1460 (Chipshop). LNP characterization was performed as described previously.[54] The resulting LNPs measured 95–120 nm with a PDI <0.2 (Figure S10) and exhibited an encapsulation efficiency of 90%–95% (data not shown).

Evaluation of transfection efficiency

The translation efficiency of the different mRNAs was evaluated by luciferase assay for the detection of NanoLuc reporter protein and by ELISA for Spike protein.

Luciferase assay

Briefly, we removed the cell media and added the Hikarazine-108 NanoLuc substrate[55] diluted in PBS buffer at a final concentration of 4 μM. Bioluminescence was then measured immediately using CLARIOstar plate reader (BMG Labtech). Relative Luciferase Units (RLUs) were normalized to protein content using BCA assay (Thermo) and expressed as RLU per mg of protein.

RBD ELISA

Briefly, the cells were harvested and lysed 24 h post-transfection, and spike protein expression was quantified using the Human SARS-CoV-2 RBD ELISA Kit (Thermo Fisher) according to the manufacturer's protocol.

Fluorescence *in situ* hybridization-flow cytometry analysis

We detected mRNA using the PrimeFlow RNA Assay Kit (Thermo Fisher) following manufacturer's protocol. We used Alexa-Fluor 488-labeled probes to detect GAPDH mRNA and Alexa Fluor 647-labeled probes to detect NanoLuc mRNA; probes were designed by Thermo Fisher scientific. Twenty thousand single-cell events were acquired for each sample on a BD FORTESSA X20 flow cytometer (Beckton Dickinson). Data were analyzed using BD FACSDiva Software (Beckton Dickinson). NanoLuc probe signal was normalized to GAPDH probe signal.

RT-qPCR

Total RNA was extracted from the samples using TRIzol reagent (Thermo Fisher) according to the manufacturer's instructions. RNA concentration and purity were measured using a NanoDrop spectrophotometer (Thermo Fisher), and the quality of the extraction was evaluated using an Agilent Bioanalyzer 2100. First-strand cDNA was synthesized from 200 ng of total RNA using the LunaScript RT SuperMix Kit (New England Biolabs).

The mRNA levels of NanoLuc, interferon alpha (IFNα/β), tumor necrosis factor alpha (TNF-α), and IL-6 were quantified at 2 and 24 h after transfection by real-time quantitative reverse-transcription polymerase chain reaction (RT-qPCR) according to our previous report.[56] For NanoLuc, the primers anneal in the first third of the gene. qPCR was performed using the Luna qPCR Master Mix (New England Biolabs) with a LightCycler 480 PCR system (Roche). The qPCR data were analyzed by the comparative ΔΔCt method, using the GAPDH RT-qPCR signal as an internal control for normalization. The primers used for RT-qPCR were synthesized by Eurogentec, and the sequences are listed in Table S4.

Polysome profiling

The translation status of different mRNAs was compared in HeLa cells using polysome profiling. One day prior to transfection, cells were seeded in 15 cm petri dishes at a density of 3×10^6 cells per dish. Transfection was performed using 10 μg of IVT mRNA complexed with Lipofectamine (LFM). Twenty-four hours post-transfection, cells were treated with 100 μg/mL cycloheximide, then harvested and lysed following a method paper.[57]

Approximately 350 μL of cell lysate was loaded onto a 10%–50% sucrose gradient. After ultracentrifugation, the gradient was fractionated into 15 distinct fractions. RNA from each fraction was isolated using TRIzol LS reagent (Thermo Fisher Scientific) according to the manufacturer's protocol. A representative polysome profile is presented in Figure S11. The isolated RNA was then reverse transcribed to cDNA, and quantitative PCR (qPCR) was performed as described in the RT-qPCR section above.

In vivo evaluation

All animal procedures were approved by the French Ministry of Research (#30279 for intramuscular injection of luciferase mRNA, #44268 for immunization with Spike mRNA).

For luciferase expression, BALB/c mice (n = 3 to 7 per group) received intramuscular (i.m.) injections of 1 μg N1-methyl-pseudouridine-modified firefly luciferase (Fluc) mRNA encapsulated in LNPs. Mice were imaged 5 min after intraperitoneal injection of 100 μL of the D-luciferin substrate (15 mg/mL) (Promega) at 6, 24, 48, and 72 h post-injection of Fluc mRNA using an IVIS Lumina Imaging System (PerkinElmer, Ville-bon-sur-Yvette, France). The

luciferase signal was quantified using Living Image software (PerkinElmer).

For the SARS-CoV-2 Spike immunization study, BALB/c mice ($n = 5$ per group) received 5 μg of N1-methyl-pseudouridine-modified Spike mRNA. LNPs were injected into the tibialis muscle on days 1 and 14. Blood was collected in Microvette 500 CAT-Gel tubes (Sarstedt) from the submandibular vein on days 0, 7, 13, and 21, then centrifuged to separate serum. Serum was stored at −80°C until assayed. Anti-Spike IgG antibodies were quantified using a mouse COVID-19 S IgG ELISA Kit (Antibodies.com). Neutralizing antibodies were quantified using an SARS-CoV-2 Surrogate Virus Neutralization Test (GenScript).

Statistical analysis

Unless otherwise noted, results are expressed as mean ± SD or SEM. Data was analyzed using Graph Pad prism 8.0 software. Comparison of significance between groups was assessed using nested one-way ANOVA.*$p < 0.05$, **$p < 0.01$, ***$p < 0.001$.

DATA AVAILABILITY

The data underlying this article are available in the article and in its online supplemental information.

ACKNOWLEDGMENTS

We thank Dr. Yves Janin (Muséum d'Histoire Naturelle, Paris) for Hikarazine NanoLuc substrate. We are grateful to Bertrand Castaing and Franck Coste for providing access to the Nano S zetasizer. We also thank Marilyne Le Mee and Stéphanie Retif, of the TAAM-In Vivo Imaging subplatforms of the MO2VING facility (Orléans, France). We are thankful to Clémence Couton who had shared her human blood sample expertise. The graphical abstract and schematics in Figures 1, 3, 4, and 7 were created with BioRender.

Ayoub Medjmedj received a PhD fellowship from Région Center Val de Loire. This work was supported by Comités Régionaux de la Ligue Nationale contre le cancer (F.P. and J.H.), CNRS Prematuration grant RNAUTR (F.P.), and by Agence Nationale de la Recherche (CREABONE Grant Number ANR-21-CE18-0037). SRCD studies were supported by Synchrotron SOLEIL and were performed under proposal 20231392 (F.P.).

AUTHOR CONTRIBUTIONS

A.M. and F.P. conceived the project and designed the experiments. A.M. performed most of the experiments. H.G. performed the bacterial tail stability and ELISA experiments. A.M., F.P., and A.N. performed plasmids cloning. J.H., F.W., F.P., and A.M. performed the SRCD experiments. C.C. performed polysomal sucrose gradient experiments. R.C. and C.G. participated in the animal experiments. D. H. and A.M. formulated and characterized the lipid nanoparticles (LNPs). A.B. established and provided the AB1079 cells. L.M. helped with MoDC. A.M. and F.P. analyzed the results, prepared the figures, and drafted the manuscript. All authors reviewed the manuscript and figures and approved the final version for submission.

DECLARATION OF INTERESTS

A.M., A.N., and F.P. are inventors of the patent WO2024231621 hold by CNRS related to the data in this work. Other authors declare no conflict of interest.

SUPPLEMENTAL INFORMATION

Supplemental information can be found online at https://doi.org/10.1016/j.omtn.2025.102648.

REFERENCES

1. Jackson, N.A.C., Kester, K.E., Casimiro, D., Gurunathan, S., and DeRosa, F. (2020). The promise of mRNA vaccines: a biotech and industrial perspective. NPJ Vaccines *5*, 11. https://doi.org/10.1038/s41541-020-0159-8.
2. Barbier, A.J., Jiang, A.Y., Zhang, P., Wooster, R., and Anderson, D.G. (2022). The clinical progress of mRNA vaccines and immunotherapies. Nat. Biotechnol. *40*, 840–854. https://doi.org/10.1038/s41587-022-01294-2.
3. Lenk, R., Kleindienst, W., Szabó, G.T., Baiersdörfer, M., Boros, G., Keller, J.M., Mahiny, A.J., and Vlatkovic, I. (2024). Understanding the impact of in vitro transcription byproducts and contaminants. Front. Mol. Biosci. *11*, 1426129. https://doi.org/10.3389/fmolb.2024.1426129.
4. Kang, D.D., Li, H., and Dong, Y. (2023). Advancements of in vitro transcribed mRNA (IVT mRNA) to enable translation into the clinics. Adv. Drug Deliv. Rev. *199*, 114961. https://doi.org/10.1016/j.addr.2023.114961.
5. Hinnebusch, A.G., Ivanov, I.P., and Sonenberg, N. (2016). Translational control by 5′-untranslated regions of eukaryotic mRNAs. Science *352*, 1413–1416. https://doi.org/10.1126/science.aad9868.
6. Bartel, D.P. (2009). MicroRNAs: target recognition and regulatory functions. Cell *136*, 215–233. https://doi.org/10.1016/j.cell.2009.01.002.
7. Mayr, C. (2019). What Are 3' UTRs Doing? Cold Spring Harbor Perspect. Biol. *11*, a034728. https://doi.org/10.1101/cshperspect.a034728.
8. Zeng, C., Hou, X., Yan, J., Zhang, C., Li, W., Zhao, W., Du, S., and Dong, Y. (2020). Leveraging mRNA Sequences and Nanoparticles to Deliver SARS-CoV-2 Antigens In Vivo. Adv. Mater. *32*, e2004452. https://doi.org/10.1002/adma.202004452.
9. Cao, J., Novoa, E.M., Zhang, Z., Chen, W.C.W., Liu, D., Choi, G.C.G., Wong, A.S.L., Wehrspaun, C., Kellis, M., and Lu, T.K. (2021). High-throughput 5' UTR engineering for enhanced protein production in non-viral gene therapies. Nat. Commun. *12*, 4138. https://doi.org/10.1038/s41467-021-24436-7.
10. Castillo-Hair, S., Fedak, S., Wang, B., Linder, J., Havens, K., Certo, M., and Seelig, G. (2024). Optimizing 5'UTRs for mRNA-delivered gene editing using deep learning. Nat. Commun. *15*, 5284. https://doi.org/10.1038/s41467-024-49508-2.
11. Sample, P.J., Wang, B., Reid, D.W., Presnyak, V., McFadyen, I.J., Morris, D.R., and Seelig, G. (2019). Human 5′ UTR design and variant effect prediction from a massively parallel translation assay. Nat. Biotechnol. *37*, 803–809. https://doi.org/10.1038/s41587-019-0164-5.
12. Orlandini von Niessen, A.G., Poleganov, M.A., Rechner, C., Plaschke, A., Kranz, L. M., Fesser, S., Diken, M., Löwer, M., Vallazza, B., Beissert, T., et al. (2019). Improving mRNA-Based Therapeutic Gene Delivery by Expression-Augmenting 3′ UTRs Identified by Cellular Library Screening. Mol. Ther. *27*, 824–836. https://doi.org/10.1016/j.ymthe.2018.12.011.
13. Xia, X. (2021). Detailed Dissection and Critical Evaluation of the Pfizer/BioNTech and Moderna mRNA Vaccines. Vaccines *9*, 734. https://doi.org/10.3390/vaccines9070734.
14. Imai, S., Kumar, P., Hellen, C.U.T., D'Souza, V.M., and Wagner, G. (2016). An accurately preorganized IRES RNA structure enables eIF4G capture for initiation of viral translation. Nat. Struct. Mol. Biol. *23*, 859–864. https://doi.org/10.1038/nsmb.3280.
15. Goldstrohm, A.C., and Wickens, M. (2008). Multifunctional deadenylase complexes diversify mRNA control. Nat. Rev. Mol. Cell Biol. *9*, 337–344. https://doi.org/10.1038/nrm2370.
16. Amrani, N., Ghosh, S., Mangus, D.A., and Jacobson, A. (2008). Translation factors promote the formation of two states of the closed-loop mRNP. Nature *453*, 1276–1280. https://doi.org/10.1038/nature06974.
17. Grier, A.E., Burleigh, S., Sahni, J., Clough, C.A., Cardot, V., Choe, D.C., Krutein, M. C., Rawlings, D.J., Jensen, M.C., Scharenberg, A.M., and Jacoby, K. (2016). pEVL: A Linear Plasmid for Generating mRNA IVT Templates With Extended Encoded Poly (A) Sequences. Mol. Ther. Nucleic Acids *5*, e306. https://doi.org/10.1038/mtna.2016.21.
18. EBERLE, F., Sahin, U., Kuhn, A., Vallazza, B., and Diken, M. (2016). Stabilization of poly(a) sequence encoding dna sequences. US10717982B2 2020-07-21.
19. Chen, H., Liu, D., Guo, J., Aditham, A., Zhou, Y., Tian, J., Luo, S., Ren, J., Hsu, A., Huang, J., et al. (2025). Branched chemically modified poly(A) tails enhance the

translation capacity of mRNA. Nat. Biotechnol. *43*, 194–203. https://doi.org/10.1038/s41587-024-02174-7.

20. Lim, J., Kim, D., Lee, Y.S., Ha, M., Lee, M., Yeo, J., Chang, H., Song, J., Ahn, K., and Kim, V.N. (2018). Mixed tailing by TENT4A and TENT4B shields mRNA from rapid deadenylation. Science *361*, 701–704. https://doi.org/10.1126/science.aam5794.
21. Chang, H., Lim, J., Ha, M., and Kim, V.N. (2014). TAIL-seq: Genome-wide Determination of Poly(A) Tail Length and 3′ End Modifications. Mol. Cell *53*, 1044–1052. https://doi.org/10.1016/j.molcel.2014.02.007.
22. Liu, Y., Nie, H., Sun, R., Wang, J., and Lu, F. (2021). Enhancement of synthetic mRNA translation efficiency through engineered poly(A) tails. Preprint at bioRxiv. https://doi.org/10.1101/2021.08.30.458298.
23. Sosnick, T.R. (2001). Characterization of Tertiary Folding of RNA by Circular Dichroism and Urea. Curr. Protoc. Nucleic Acid Chem. *Chapter 11*, Unit 11.5. https://doi.org/10.1002/0471142700.nc1105s04.
24. Kloczewiak, M., Banks, J.M., Jin, L., and Brader, M.L. (2022). A Biopharmaceutical Perspective on Higher-Order Structure and Thermal Stability of mRNA Vaccines. Mol. Pharm. *19*, 2022–2031. https://doi.org/10.1021/acs.molpharmaceut.2c00092.
25. Wien, F., Geinguenaud, F., Grange, W., and Arluison, V. (2021). SRCD and FTIR Spectroscopies to Monitor Protein-Induced Nucleic Acid Remodeling. In RNA Remodeling Proteins: Methods and Protocols, M. Boudvillain, ed. (Springer US), pp. 87–108. https://doi.org/10.1007/978-1-0716-0935-4_6.
26. Vivinus, S., Baulande, S., van Zanten, M., Campbell, F., Topley, P., Ellis, J.H., Dessen, P., and Coste, H. (2001). An element within the 5′ untranslated region of human Hsp70 mRNA which acts as a general enhancer of mRNA translation. Eur. J. Biochem. *268*, 1908–1917. https://doi.org/10.1046/j.1432-1327.2001.02064.x.
27. Linares-Fernández, S., Moreno, J., Lambert, E., Mercier-Gouy, P., Vachez, L., Verrier, B., and Exposito, J.-Y. (2021). Combining an optimized mRNA template with a double purification process allows strong expression of in vitro transcribed mRNA. Mol. Ther. Nucleic Acids *26*, 945–956. https://doi.org/10.1016/j.omtn.2021.10.007.
28. Licursi, M., Christian, S.L., Pongnopparat, T., and Hirasawa, K. (2011). In vitro and in vivo comparison of viral and cellular internal ribosome entry sites for bicistronic vector expression. Gene Ther. *18*, 631–636. https://doi.org/10.1038/gt.2011.11.
29. Gross, L., Vicens, Q., Einhorn, E., Noireterre, A., Schaeffer, L., Kuhn, L., Imler, J.-L., Eriani, G., Meignin, C., and Martin, F. (2017). The IRES5′UTR of the dicistrovirus cricket paralysis virus is a type III IRES containing an essential pseudoknot structure. Nucleic Acids Res. *45*, 8993–9004. https://doi.org/10.1093/nar/gkx622.
30. Godet, A.-C., David, F., Hantelys, F., Tatin, F., Lacazette, E., Garmy-Susini, B., and Prats, A.-C. (2019). IRES Trans-Acting Factors, Key Actors of the Stress Response. Int. J. Mol. Sci. *20*, 924. https://doi.org/10.3390/ijms20040924.
31. Rubtsova, M.P., Sizova, D.V., Dmitriev, S.E., Ivanov, D.S., Prassolov, V.S., and Shatsky, I.N. (2003). Distinctive Properties of the 5′-Untranslated Region of Human Hsp70 mRNA. J. Biol. Chem. *278*, 22350–22356. https://doi.org/10.1074/jbc.M303213200.
32. Yang, A.-D., Barro, M., Gorziglia, M.I., and Patton, J.T. (2004). Translation enhancer in the 3'-untranslated region of rotavirus gene 6 mRNA promotes expression of the major capsid protein VP6. Arch. Virol. *149*, 303–321. https://doi.org/10.1007/s00705-003-0211-9.
33. Chung, D.J., Wright, A.E., and Clerch, L.B. (1998). The 3' untranslated region of manganese superoxide dismutase RNA contains a translational enhancer element. Biochemistry *37*, 16298–16306. https://doi.org/10.1021/bi980935g.
34. Kuhn, A.N., Diken, M., Kreiter, S., Vallazza, B., Türeci, Ö., and Sahin, U. (2011). Determinants of intracellular RNA pharmacokinetics: Implications for RNA-based immunotherapeutics. RNA Biol. *8*, 35–43. https://doi.org/10.4161/rna.8.1.13767.
35. Uchida, S., Perche, F., Pichon, C., and Cabral, H. (2020). Nanomedicine-Based Approaches for mRNA Delivery. Mol. Pharm. *17*, 3654–3684. https://doi.org/10.1021/acs.molpharmaceut.0c00618.
36. Dmitriev, S.E., Andreev, D.E., Adyanova, Z.V., Terenin, I.M., and Shatsky, I.N. (2009). Efficient cap-dependent translation of mammalian mRNAs with long and highly structured 5′-untranslated regions in vitro and in vivo. Mol. Biol. *43*, 108–113. https://doi.org/10.1134/S0026893309010154.
37. Warminski, M., Mamot, A., Depaix, A., Kowalska, J., and Jemielity, J. (2023). Chemical Modifications of mRNA Ends for Therapeutic Applications. Acc. Chem. Res. *56*, 2814–2826. https://doi.org/10.1021/acs.accounts.3c00442.
38. Sahin, U., Muik, A., Derhovanessian, E., Vogler, I., Kranz, L.M., Vormehr, M., Baum, A., Pascal, K., Quandt, J., Maurus, D., et al. (2020). COVID-19 vaccine BNT162b1 elicits human antibody and TH1 T cell responses. Nature *586*, 594–599. https://doi.org/10.1038/s41586-020-2814-7.
39. Svitkin, Y.V., Cheng, Y.M., Chakraborty, T., Presnyak, V., John, M., and Sonenberg, N. (2017). N1-methyl-pseudouridine in mRNA enhances translation through eIF2α-dependent and independent mechanisms by increasing ribosome density. Nucleic Acids Res. *45*, 6023–6036. https://doi.org/10.1093/nar/gkx135.
40. Arrigucci, R., Bushkin, Y., Radford, F., Lakehal, K., Vir, P., Pine, R., Martin, D., Sugarman, J., Zhao, Y., Yap, G.S., et al. (2017). FISH-Flow, a protocol for the concurrent detection of mRNA and protein in single cells using fluorescence in situ hybridization and flow cytometry. Nat. Protoc. *12*, 1245–1260. https://doi.org/10.1038/nprot.2017.039.
41. Lee, Y.S., Levdansky, Y., Jung, Y., Kim, V.N., and Valkov, E. (2024). Deadenylation kinetics of mixed poly(A) tails at single-nucleotide resolution. Nat. Struct. Mol. Biol. *31*, 826–834. https://doi.org/10.1038/s41594-023-01187-1.
42. Weingarten-Gabbay, S., Elias-Kirma, S., Nir, R., Gritsenko, A.A., Stern-Ginossar, N., Yakhini, Z., Weinberger, A., and Segal, E. (2016). Systematic discovery of cap-independent translation sequences in human and viral genomes. Science *351*, aad4939. https://doi.org/10.1126/science.aad4939.
43. Gebauer, F., and Hentze, M.W. (2016). IRES unplugged. Science *351*, 228. https://doi.org/10.1126/science.aad8540.
44. Kirshina, A., Vasileva, O., Kunyk, D., Seregina, K., Muslimov, A., Ivanov, R., and Reshetnikov, V. (2023). Effects of Combinations of Untranslated-Region Sequences on Translation of mRNA. Biomolecules *13*, 1677. https://doi.org/10.3390/biom13111677.
45. Bicknell, A.A., Reid, D.W., Licata, M.C., Jones, A.K., Cheng, Y.M., Li, M., Hsiao, C.J., Pepin, C.S., Metkar, M., Levdansky, Y., et al. (2024). Attenuating ribosome load improves protein output from mRNA by limiting translation-dependent mRNA decay. Cell Rep. *43*, 114098. https://doi.org/10.1016/j.celrep.2024.114098.
46. Lewis, C.J.T., Xie, L.H., Bhandarkar, S.M., Jin, D., Abdallah, K., Draycott, A.S., Chen, Y., Thoreen, C.C., and Gilbert, W.V. (2025). Quantitative profiling of human translation initiation reveals elements that potently regulate endogenous and therapeutically modified mRNAs. Mol. Cell *85*, 445–459.e5. https://doi.org/10.1016/j.molcel.2024.11.030.
47. Petersen, C.P., Bordeleau, M.-E., Pelletier, J., and Sharp, P.A. (2006). Short RNAs Repress Translation after Initiation in Mammalian Cells. Mol. Cell *21*, 533–542. https://doi.org/10.1016/j.molcel.2006.01.031.
48. Burman, L.G., and Mauro, V.P. (2012). Analysis of rRNA processing and translation in mammalian cells using a synthetic 18S rRNA expression system. Nucleic Acids Res. *40*, 8085–8098. https://doi.org/10.1093/nar/gks530.
49. Hall, M.P., Unch, J., Binkowski, B.F., Valley, M.P., Butler, B.L., Wood, M.G., Otto, P., Zimmerman, K., Vidugiris, G., Machleidt, T., et al. (2012). Engineered Luciferase Reporter from a Deep Sea Shrimp Utilizing a Novel Imidazopyrazinone Substrate. ACS Chem. Biol. *7*, 1848–1857. https://doi.org/10.1021/cb3002478.
50. Mathur, L., Jung, S., Jang, C., and Lee, G. (2021). Quantitative analysis of m6A RNA modification by LC-MS. STAR Protoc. *2*, 100724. https://doi.org/10.1016/j.xpro.2021.100724.
51. Miles, A.J., and Wallace, B.A. (2018). CDtoolX, a downloadable software package for processing and analyses of circular dichroism spectroscopic data. Protein Sci. *27*, 1717–1722. https://doi.org/10.1002/pro.3474.
52. Mamchaoui, K., Trollet, C., Bigot, A., Negroni, E., Chaouch, S., Wolff, A., Kandalla, P.K., Marie, S., Di Santo, J., St Guily, J.L., et al. (2011). Immortalized pathological human myoblasts: towards a universal tool for the study of neuromuscular disorders. Skelet. Muscle *1*, 34. https://doi.org/10.1186/2044-5040-1-34.
53. Shen, Z., Reznikoff, G., Dranoff, G., and Rock, K.L. (1997). Cloned dendritic cells can present exogenous antigens on both MHC class I and class II molecules. J. Immunol. *158*, 2723–2730.
54. Medjmedj, A., Ngalle-Loth, A., Clemençon, R., Hamacek, J., Pichon, C., and Perche, F. (2022). Cellulo and In Vivo Comparison of Cholesterol,

Beta-Sitosterol and Dioleylphosphatidylethanolamine for Lipid Nanoparticle Formulation of mRNA. Nanomater. Basel Switz. *12*, 2446. https://doi.org/10.3390/nano12142446.

55. Coutant, E.P., Goyard, S., Hervin, V., Gagnot, G., Baatallah, R., Jacob, Y., Rose, T., and Janin, Y.L. (2019). Gram-scale synthesis of luciferins derived from coelenterazine and original insights into their bioluminescence properties. Org. Biomol. Chem. *17*, 3709–3713. https://doi.org/10.1039/C9OB00459A.

56. Wang, P., Perche, F., Midoux, P., Cabral, C.S.D., Malard, V., Correia, I.J., Ei-Hafci, H., Petite, H., Logeart-Avramoglou, D., and Pichon, C. (2021). In Vivo bone tissue induction by freeze-dried collagen-nanohydroxyapatite matrix loaded with BMP2/NS1 mRNAs lipopolyplexes. J. Contr. Release *334*, 188–200. https://doi.org/10.1016/j.jconrel.2021.04.021.

57. Morita, M., Alain, T., Topisirovic, I., and Sonenberg, N. (2013). Polysome Profiling Analysis. Bio. Protoc. *3*. https://doi.org/10.21769/BioProtoc.833.

OMTN, Volume *36*

Supplemental information

Evaluation of synthetic mRNA with selected UTR sequences and alternative poly(A) tail, *in vitro* and *in vivo*

Ayoub Medjmedj, Hugo Genon, Dounia Hezili, Albert Ngalle Loth, Rudy Clemençon, Cyril Guimpied, Lucile Mollet, Anne Bigot, Frank Wien, Josef Hamacek, Clément Chapat, and Federico Perche

Table S1: *sequences of the poly(A) tails evaluated.*

Name	Sequence
A120	AAAAAAAAAAAAAAAAAAAAAAAAAAAAAAAAAAAAAAAAAAAAAAAAAAAAAAAAAAAAA<br>AAAAAAAAAAAAAAAAAAAAAAAAAAAAAAAAAAAAAAAAAAAAAAAAAAAAAAAAAAA
A30L70	AAAAAAAAAAAAAAAAAAAAAAAAAAAAAAGCATATGACTAAAAAAAAAAAAAAAAAAAAAA<br>AAAAAAAAAAAAAAAAAAAAAAAAAAAAAAAAAAAAAAAAAAAAAAAAAA
A/G	AAAAAAAAAAGAAAAAAAAAAGAAAAAAAAAAGAAAAAAAAAAGAAAAAAAAAAGAAAAAA<br>AAAAGAAAAAAAAAAGAAAAAAAAAAGAAAAAAAAAAGAAAAAAAAAAGAAAAAAAAAAGA<br>AAAAAAAAAG

Table S2: *dsRNA levels of different IVT mRNA: All mRNAs are capped with ARCA, except for the Oz Bioscience mRNA, which is cap1.*

5'UTR	CDS	3'UTR	dsRNA % ± SD
hAg	Nano luciferase	AES	0.361 ± 0.144
		VP6	0.510 ± 0.219
		SOD	0.280 ± 0.013
UTR3		AES	0.294 ± 0.072
		VP6	0.233 ± 0.020
		SOD	0.209 ± 0.011
UTR4		AES	0.331 ± 0.147
		VP6	0.291 ± 0.079
		SOD	0.206 ± 0.008
hAg	Firefly luciferase	AES	0.431 ± 0.117
		VP6	0.379 ± 0.184
		SOD	0.259 ± 0.025
UTR3		AES	0.239 ± 0.003
		VP6	0.293 ± 0.014
		SOD	0.302 ± 0.105
UTR4		AES	0.263 ± 0.028
		VP6	0.407 ± 0.095
		SOD	0.276 ± 0.006
Firefly luciferase Oz Bioscience			0.196 ± 0.036
hAg	Spike	AES	0.175 ± 0.018
		VP6	0.163 ± 0.024
		SOD	0.142 ± 0.004

***Table S3**: dsRNA level of IVT mRNA using different cap, U or m1Ψ, and before/after oligo dT purification.*

Cap	Nucleotides	dsRNA % ± SD<br>Without Oligo dT purification	 With Oligo dT purification
None	U	0.365 ± 0.063	0.154 ± 0.008
	m1Ψ	0.288 ± 0.115	0.129 ± 0.013
ARCA	U	0.947 ± 0.112	0.552 ± 0.144
	m1Ψ	0.112 ± 0.004	0.118 ± 0.013
CleanCap AG	U	0.544 ± 0.089	0.247 ± 0.015
	m1Ψ	0.105 ± 0.004	0.140 ± 0.014

***Table S4**: qPCR primers*

	Sequences (5' -> 3')<br>FW	 RV
IFN-α	AAGCCATCCTTGTGCTAAGAGA	AGCAAGTTGGTTGAGGAAGAGA
IFN-β	AGCTCCAAGAAAGGACGAACA	GCCCTGTAGGTGAGGTTGAT
TNF-α	ATGAGCACAGAAAGCATGA	AGTAGACAGAAGAGCGTGGT
IL-6	CCTCTGGTCTTCTGGAGTACC	ACTCCTTCTGTGACTCCAGC
Nluc	GGAGGTGTGTCCAGTTTGTTTCAG	CACAGGGTACACCACCTTAA
GAPDH	GCTGCGTTTTACACCCTTTC	GTTTGCTCCACCCAACTGC

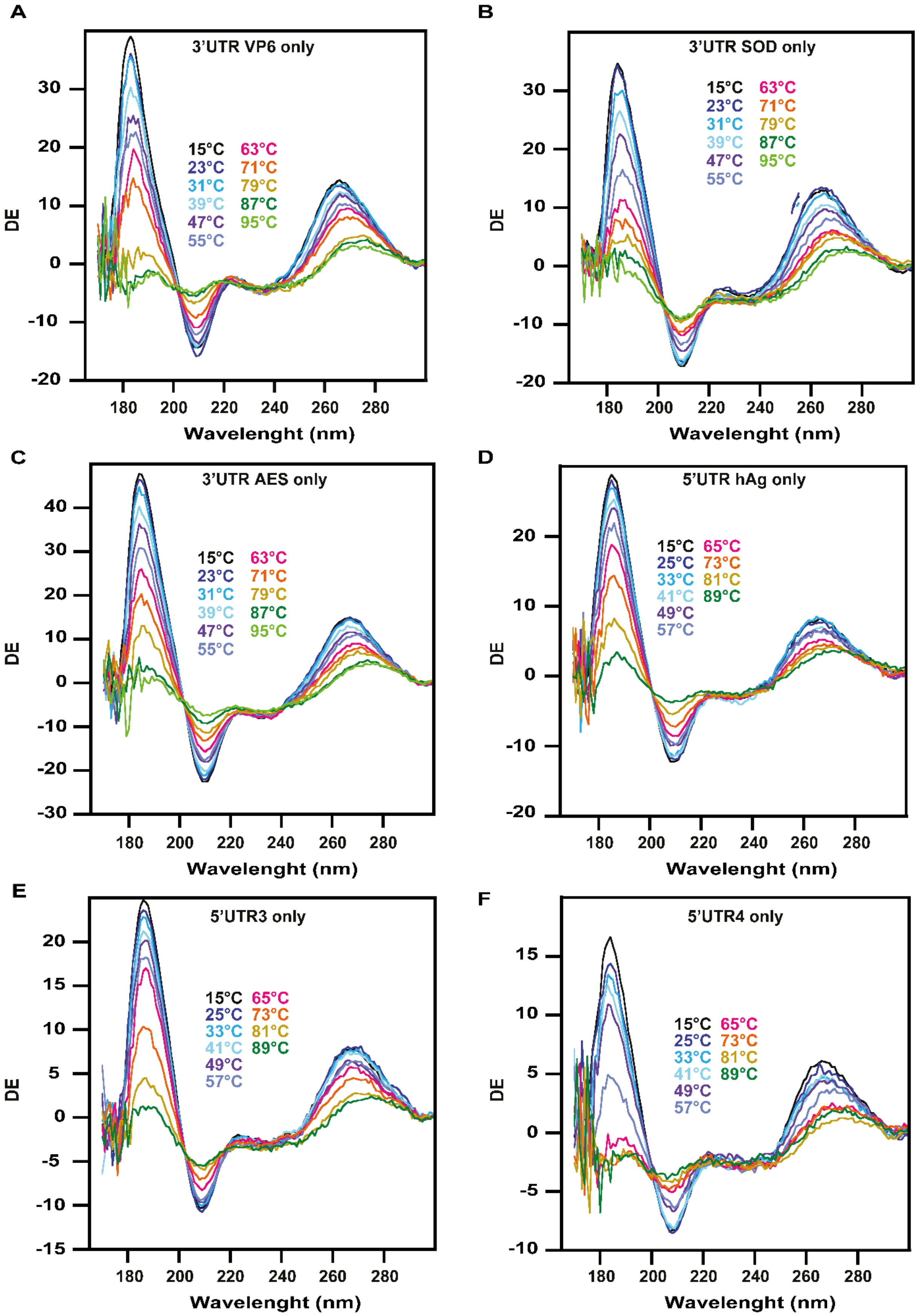

Figure S1: *Synchrotron Radiation Circular Dichroism analysis of UTR sequences. (A) 3'UTR VP6, (B) 3'UTR SOD, (C) 3'UTR AES, (D) 5'UTR hAg, (E) 5'UTR3, (F) 5'UTR4. DE: Delta Epsilon*

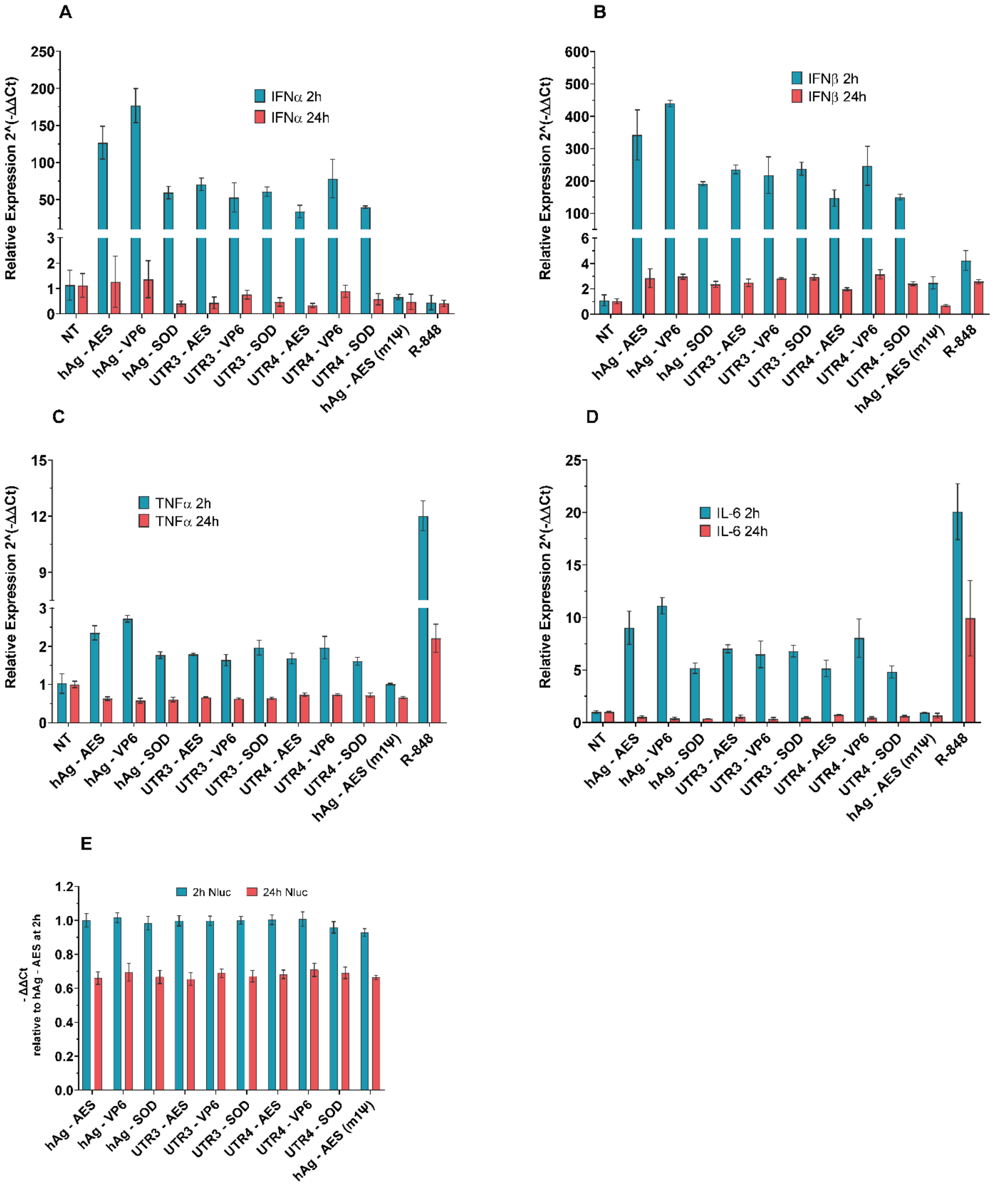

Figure S2: *qPCR results of the immunogenicity of the IVT mRNA in DC 2.4 after transfection. Results show moderate and transient activation of the interferon pathway at 2h post transfection before returning to basal levels at 24h post transfection.*

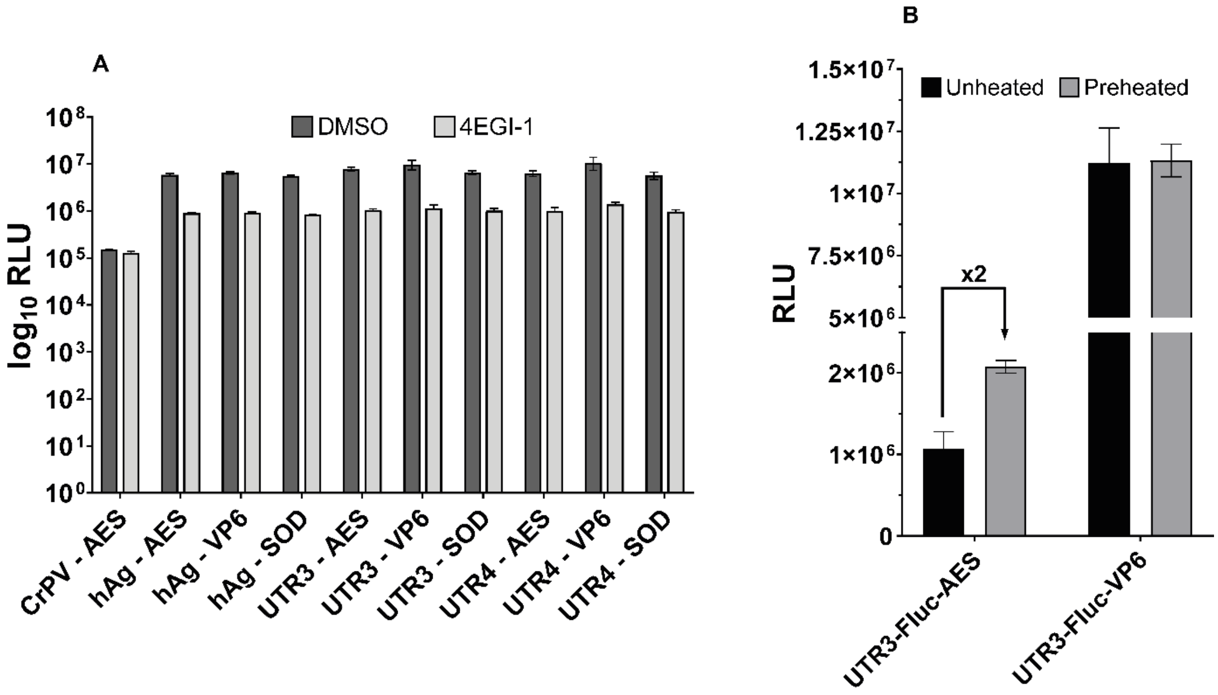

Figure S3: *In vitro translation in the rabbit reticulocytes lysate (RRL): A) Nano luciferase B) Firefly luciferase*

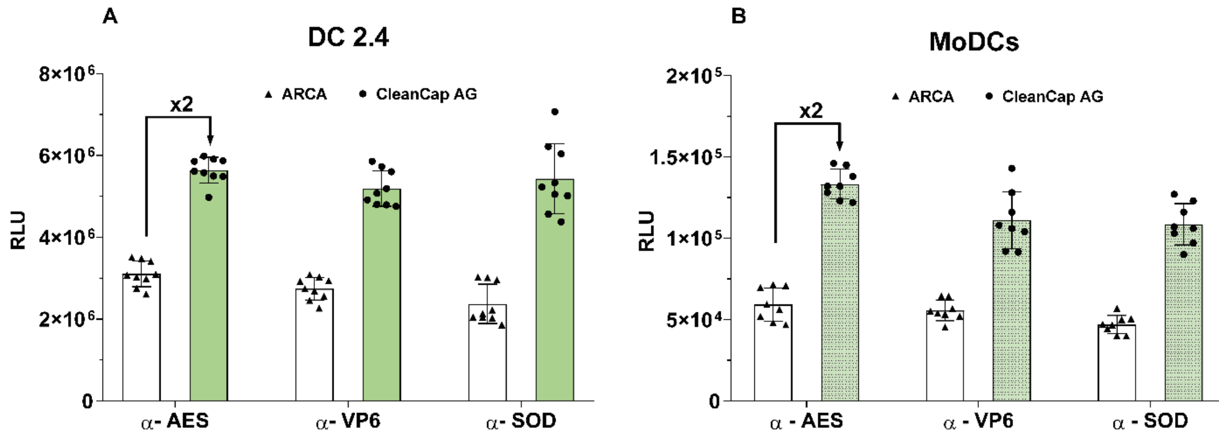

Figure S4: *ARCA-capped mRNA compared to CleanCap AG-capped mRNA in DC 2.4 cells and MoDCs primary cells*

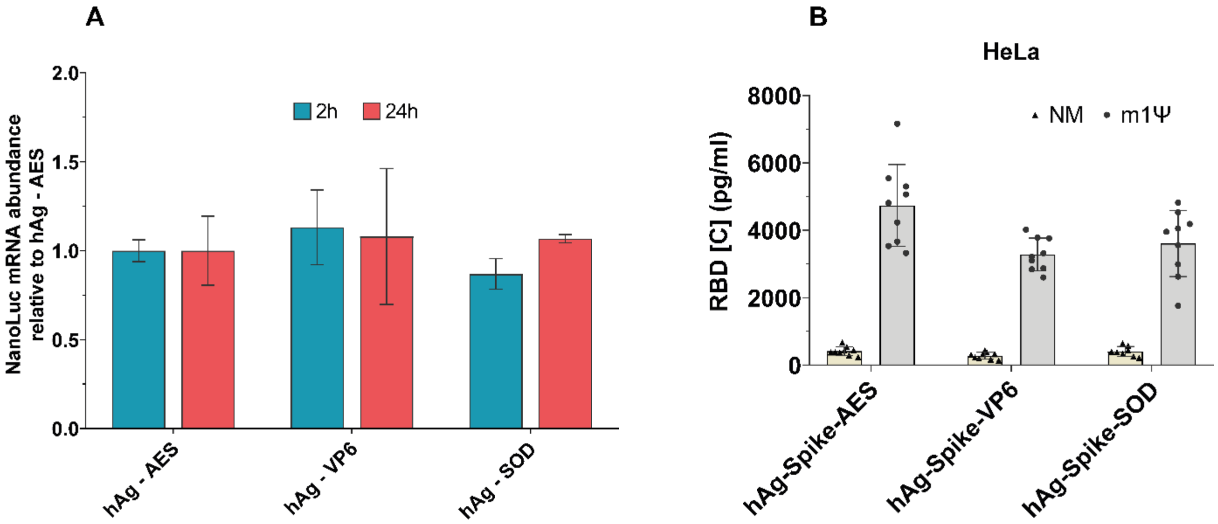

Figure S5: *A) FISH-Flow results of Nano luciferase mRNA, B) non modified spike mRNA compared to m1Ψ spike mRNA in HeLa cells 24 hours after transfection.*

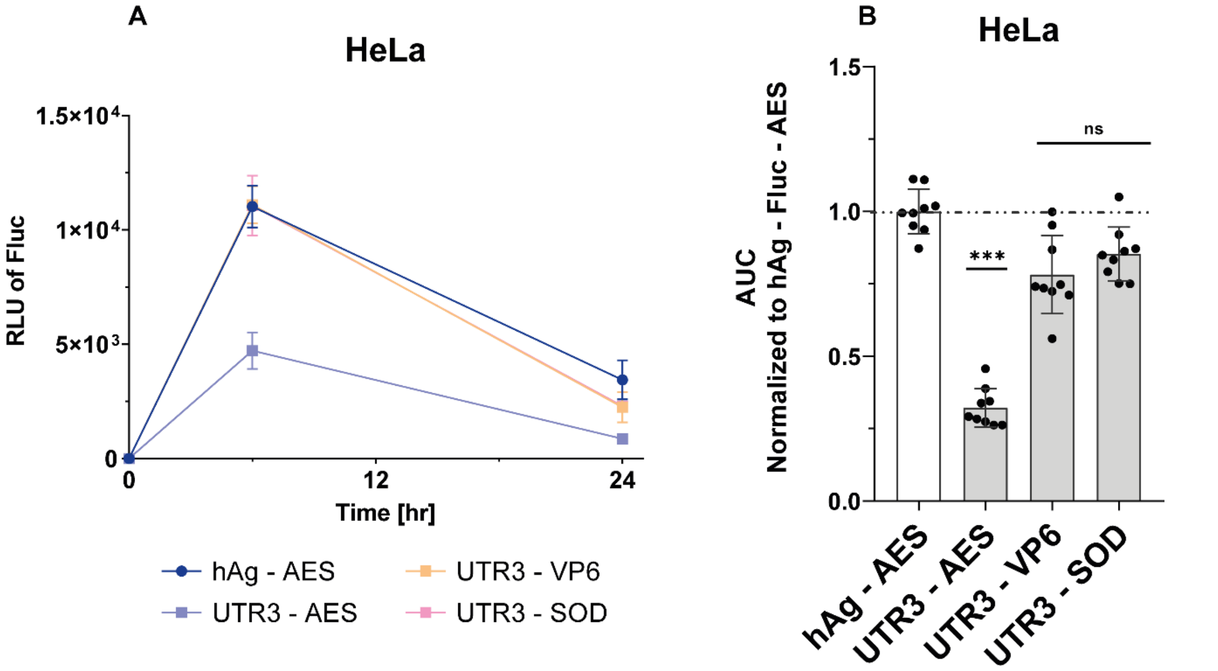

Figure S6: *Evaluation of 5'UTR3 combinations in HeLa cells: (A) Time course analysis of Fluc expression in HeLa cells transfected with the three mRNA constructs and the benchmark mRNA. Bioluminescence was acquired at 6, and 24h post-transfection, and results are expressed as mean ± SD. (B) Total protein expression over time (area under the curve) calculated from time course data and normalized to the hAg 5'UTR-AES 3'UTR.*

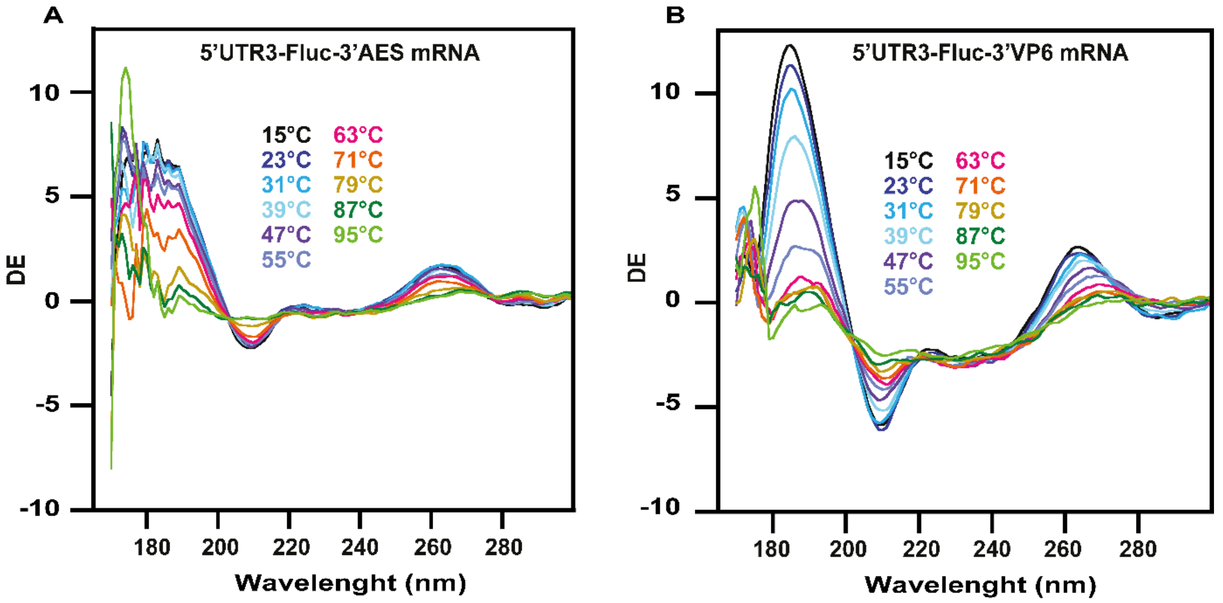

Figure S7: *Synchrotron Radiation Circular Dichroism analysis of (A) 5'UTR3-Fluc-3'AES mRNA and (B) 5'UTR3-Fluc-3'VP6 mRNA. DE: Delta Epsilon*

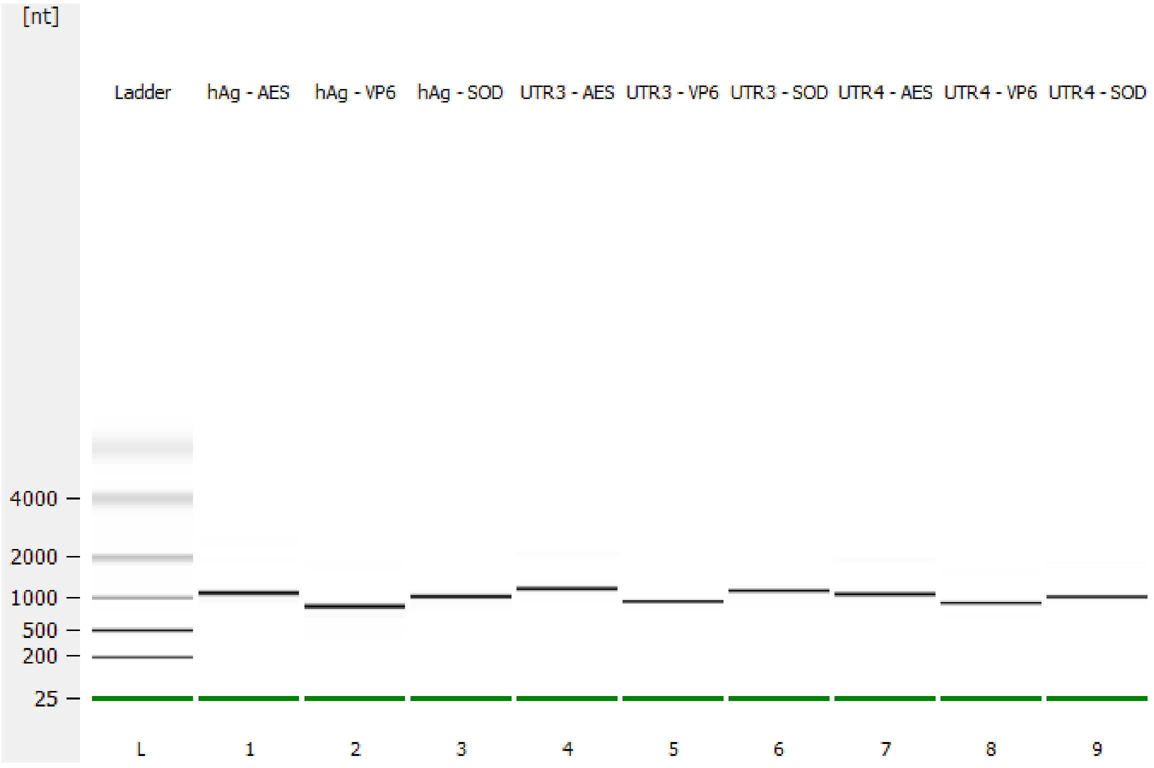

***Figure S8**: Agilent gel of IVT ARCA Nano luciferase mRNA. We confirm the good quality of our mRNAs.*

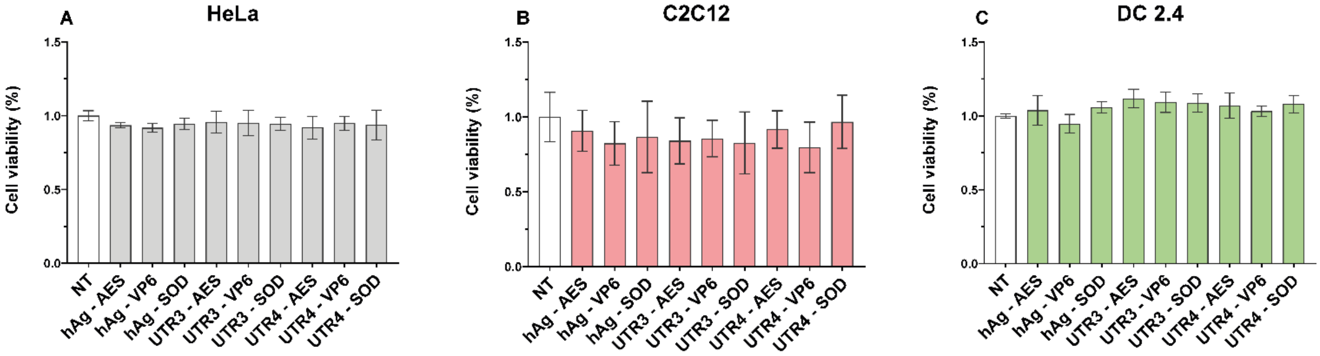

***Figure S9:** Cell viability 24 hours after transfection with Lipofectamine MessengerMAX complexes as determined by the MTT assay.*

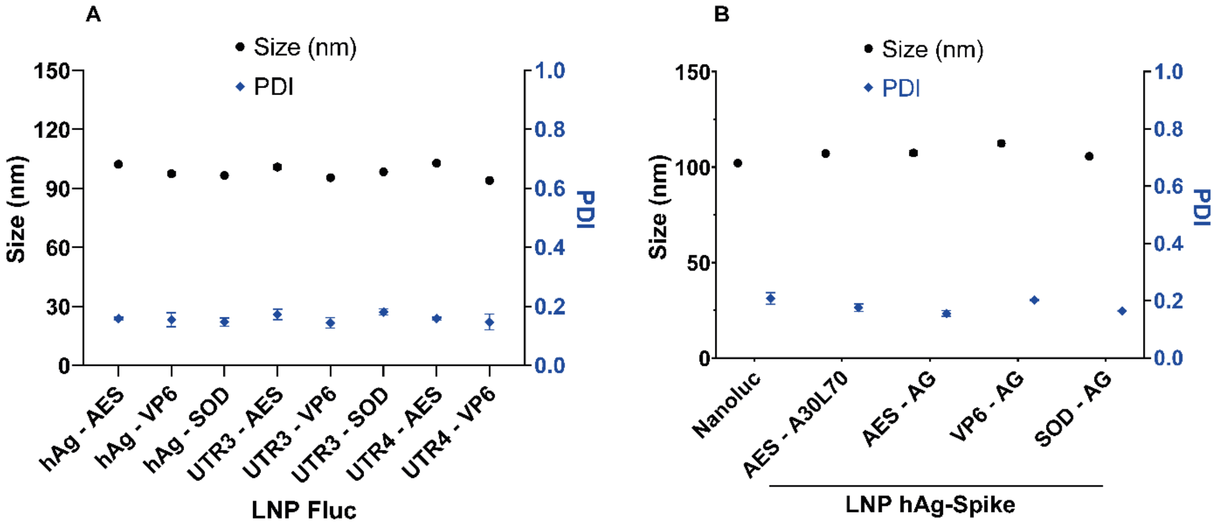

Figure S10: *LNP characterization: A) Fluc LNP, B) Spike LNP.*

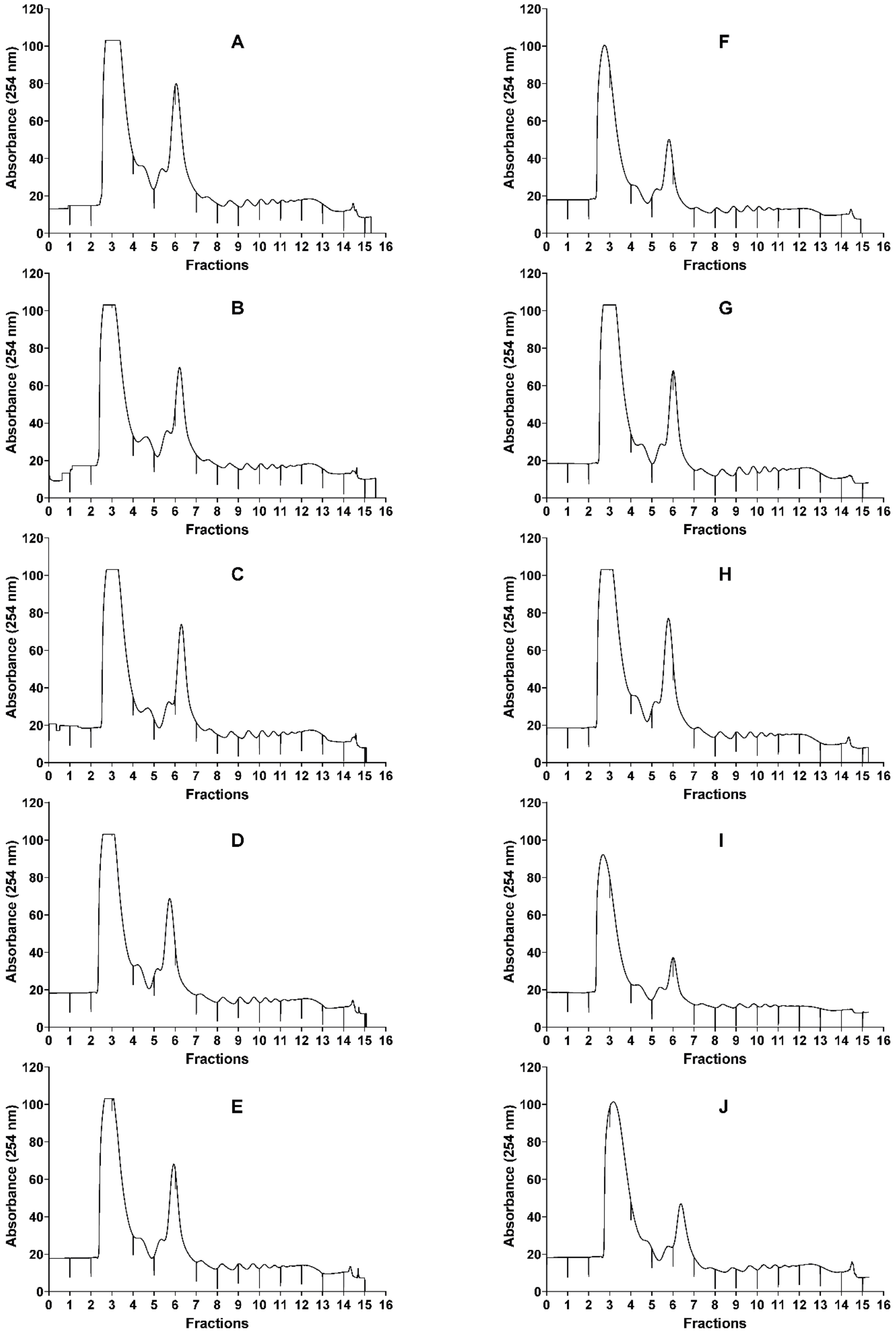

Figure S11: *Polysomes Profiles 24h after transfection: A) NT, B) hAg-AES, C) hAg-VP6, D) hAg-SOD, E) UTR3-AES, F) UTR3-VP6, G) UTR3-SOD, H) UTR4-AES, I) UTR4-VP6, J) UTR4-SOD*
